# Hormonal Contraception and the Risk of HIV Acquisition: An Individual Participant Data Meta-analysis

**DOI:** 10.1371/journal.pmed.1001778

**Published:** 2015-01-22

**Authors:** Charles S. Morrison, Pai-Lien Chen, Cynthia Kwok, Jared M. Baeten, Joelle Brown, Angela M. Crook, Lut Van Damme, Sinead Delany-Moretlwe, Suzanna C. Francis, Barbara A. Friedland, Richard J. Hayes, Renee Heffron, Saidi Kapiga, Quarraisha Abdool Karim, Stephanie Karpoff, Rupert Kaul, R. Scott McClelland, Sheena McCormack, Nuala McGrath, Landon Myer, Helen Rees, Ariane van der Straten, Deborah Watson-Jones, Janneke H. H. M. van de Wijgert, Randy Stalter, Nicola Low

**Affiliations:** 1 Clinical Sciences, FHI 360, Durham, North Carolina, United States of America; 2 Biostatistics, FHI 360, Durham, North Carolina, United States of America; 3 Department of Global Health, Medicine, and Epidemiology, University of Washington, Seattle, Washington, United States of America; 4 Department of Epidemiology, University of California, Los Angeles, Los Angeles, California, United States of America; 5 Medical Research Council, Comprehensive Clinical Trials Unit at UCL, University College London, London, United Kingdom; 6 Department of Global Health, Bill & Melinda Gates Foundation, Seattle, Washington, United States of America; 7 Wits Reproductive Health and HIV Institute, Johannesburg, South Africa; 8 Department of Infectious Disease Epidemiology, London School of Hygiene & Tropical Medicine, London, United Kingdom; 9 Population Council, New York, New York, United States of America; 10 Center for the AIDS Program of Research in South Africa, University of Kwa-Zulu Natal, Durban, South Africa; 11 Diversity Research Programs, Multicenter Protocols Group, Memorial Sloan-Kettering Cancer Center, New York, New York, United States of America; 12 Department of Medicine, University of Toronto, Toronto, Ontario, Canada; 13 Department of Social Statistics and Demography, Academic Unit of Primary Care, Population Sciences, University of Southampton, Southampton, United Kingdom; 14 Division of Epidemiology and Biostatistics, School of Public Health and Family Medicine, University of Cape Town, Cape Town, South Africa; 15 Women’s Global Health Imperative, RTI International, San Francisco, California, United States of America; 16 Department of Clinical Research, London School of Hygiene & Tropical Medicine, London, United Kingdom; 17 Department of Clinical Infection, Microbiology and Immunology, Institute of Infection and Global Health, University of Liverpool, Merseyside, United Kingdom; 18 Institute of Social and Preventive Medicine, University of Bern, Bern, Switzerland; John Hopkins University, UNITED STATES

## Abstract

**Background:**

Observational studies of a putative association between hormonal contraception (HC) and HIV acquisition have produced conflicting results. We conducted an individual participant data (IPD) meta-analysis of studies from sub-Saharan Africa to compare the incidence of HIV infection in women using combined oral contraceptives (COCs) or the injectable progestins depot-medroxyprogesterone acetate (DMPA) or norethisterone enanthate (NET-EN) with women not using HC.

**Methods and Findings:**

Eligible studies measured HC exposure and incident HIV infection prospectively using standardized measures, enrolled women aged 15–49 y, recorded ≥15 incident HIV infections, and measured prespecified covariates. Our primary analysis estimated the adjusted hazard ratio (aHR) using two-stage random effects meta-analysis, controlling for region, marital status, age, number of sex partners, and condom use. We included 18 studies, including 37,124 women (43,613 woman-years) and 1,830 incident HIV infections. Relative to no HC use, the aHR for HIV acquisition was 1.50 (95% CI 1.24–1.83) for DMPA use, 1.24 (95% CI 0.84–1.82) for NET-EN use, and 1.03 (95% CI 0.88–1.20) for COC use. Between-study heterogeneity was mild (I^2^ < 50%). DMPA use was associated with increased HIV acquisition compared with COC use (aHR 1.43, 95% CI 1.23–1.67) and NET-EN use (aHR 1.32, 95% CI 1.08–1.61). Effect estimates were attenuated for studies at lower risk of methodological bias (compared with no HC use, aHR for DMPA use 1.22, 95% CI 0.99–1.50; for NET-EN use 0.67, 95% CI 0.47–0.96; and for COC use 0.91, 95% CI 0.73–1.41) compared to those at higher risk of bias (p_interaction_ = 0.003). Neither age nor herpes simplex virus type 2 infection status modified the HC–HIV relationship.

**Conclusions:**

This IPD meta-analysis found no evidence that COC or NET-EN use increases women’s risk of HIV but adds to the evidence that DMPA may increase HIV risk, underscoring the need for additional safe and effective contraceptive options for women at high HIV risk. A randomized controlled trial would provide more definitive evidence about the effects of hormonal contraception, particularly DMPA, on HIV risk.

## Abstract 2

In a meta-analysis of individual participant data, Charles Morrison and colleagues explore the association between hormonal contraception use and risk of HIV infection in sub-Saharan Africa.

## Editors’ Summary

**Background:**

AIDS has killed about 36 million people since the first recorded case of the disease in 1981. About 35 million people (including 25 million living in sub-Saharan Africa) are currently infected with HIV, the virus that causes AIDS, and every year, another 2.3 million people become newly infected with HIV. At the beginning of the epidemic, more men than women were infected with HIV. Now, about half of all adults infected with HIV are women. In 2013, almost 60% of all new HIV infections among young people aged 15–24 years occurred among women, and it is estimated that, worldwide, 50 young women are newly infected with HIV every hour. Most women become infected with HIV through unprotected intercourse with an infected male partner—biologically, women are twice as likely to become infected through unprotected intercourse as men. A woman’s risk of becoming infected with HIV can be reduced by abstaining from sex, by having one or a few partners, and by always using condoms.

**Why Was This Study Done?:**

Women and societies both benefit from effective contraception. When contraception is available, women can avoid unintended pregnancies, fewer women and babies die during pregnancy and childbirth, and maternal and infant health improves. However, some (but not all) observational studies (investigations that measure associations between the characteristics of participants and their subsequent development of specific diseases) have reported an association between hormonal contraceptive use and an increased risk of HIV acquisition by women. So, does hormonal contraception increase the risk of HIV acquisition among women or not? Here, to investigate this question, the researchers undertake an individual participant data meta-analysis of studies conducted in sub-Saharan Africa (a region where both HIV infection and unintended pregnancies are common) to compare the incidence of HIV infection (the number of new cases in a population during a given time period) among women using and not using hormonal contraception. Meta-analysis is a statistical method that combines the results of several studies; an individual participant data meta-analysis combines the data recorded for each individual involved in the studies rather than the aggregated results from each study.

**What Did the Researchers Do and Find?:**

The researchers included 18 studies that measured hormonal contraceptive use and incident HIV infection among women aged 15–49 years living in sub-Saharan Africa in their meta-analysis. More than 37,000 women took part in these studies, and 1,830 became newly infected with HIV. Half of the women were not using hormonal contraception, a quarter were using depot-medroxyprogesterone acetate (DMPA; an injectable hormonal contraceptive), and the remainder were using combined oral contraceptives (COCs) or norethisterone enanthate (NET-EN, another injectable contraceptive). After adjustment for other factors likely to influence HIV acquisition (for example, condom use), women using DMPA had a 1.5-fold increased risk of HIV acquisition compared to women not using hormonal contraception. There was a slightly increased risk of HIV acquisition among women using NET-EN compared to women not using hormonal contraception, but this increase was not statistically significant (it may have happened by chance alone). There was no increased risk of HIV acquisition associated with COC use. DMPA use was associated with a 1.43-fold and 1.32-fold increased risk of HIV acquisition compared with COC and NET-EN use, respectively. Finally, neither age nor herpes simplex virus 2 infection status modified the effect of hormonal contraceptive use on HIV acquisition.

**What Do These Findings Mean?:**

The findings of this individual patient data meta-analysis provide no evidence that COC or NET-EN use increases a woman’s risk of acquiring HIV, but add to the evidence suggesting that DMPA use increases the risk of HIV acquisition. These findings are likely to be more accurate than those of previous meta-analyses that used aggregated data but are likely to be limited by the quality, design, and representativeness of the studies included in the analysis. These findings nevertheless highlight the need to develop additional safe and effective contraceptive options for women at risk of HIV, particularly those living in sub-Saharan Africa, where although contraceptive use is generally low, DMPA is the most widely used hormonal contraceptive. In addition, these findings highlight the need to initiate randomized controlled trials to provide more definitive evidence of the effects of hormonal contraception, particularly DMPA, on HIV risk.

**Additional Information.:**

Please access these websites via the online version of this summary at http://dx.doi.org/10.1371/journal.pmed.1001778.

- Information is available from the US National Institute of Allergy and Infectious Diseases on HIV infection and AIDS
- NAM/aidsmap provides basic information about HIV/AIDS, and summaries of recent research findings on HIV care and treatment, including personal stories about living with HIV/AIDS and a news report on this meta-analysis
- Information is available from Avert, an international AIDS charity, on many aspects of HIV/AIDS, including detailed information on women, HIV, and AIDS, and on HIV and AIDS in South Africa (in English and Spanish); personal stories of women living with HIV are available
- The World Health Organization provides information on all aspects of HIV/AIDS (in several languages); information about a 2012 WHO technical consultation about hormonal contraception and HIV
- The 2013 UNAIDS World AIDS Day report provides up-to-date information about the AIDS epidemic and efforts to halt it; UNAIDS also provides information about HIV and hormonal contraception

## Introduction

There is ongoing debate whether hormonal contraception (HC) increases the risk of HIV acquisition [1–4]. Strong evidence for an association would have important implications for sexual and reproductive health, particularly in areas of sub-Saharan Africa where the incidence of both HIV infection and unintended pregnancy remain high [5–7]. Contraception has profound benefits for women and societies, including reduced maternal and infant mortality and morbidity, empowerment of women to make choices about fertility, associated economic improvement, and a reduction in the number of babies born with HIV [8]. Although contraceptive prevalence remains low in much of sub-Saharan Africa, combined oral contraceptives (COCs, containing both estrogen and progestin) and the injectable progestins depot-medroxyprogesterone acetate (DMPA, given every 3 mo) and norethisterone enanthate (NET-EN, given every 2 mo) are the most popular contraceptive methods [9], with DMPA being the most commonly used method overall.

HC, particularly DMPA, has been reported to be associated with increased risk of HIV acquisition in some, but not all, studies [1–4]. Such a relationship is biologically plausible based on laboratory, animal, and human data [1, 10]. However, many individual studies have important methodological flaws, including lack of accurate measurement of hormonal contraceptive exposures, failure to control for important confounding factors, poor follow-up, and small sample sizes [2, 3]. A systematic review of studies published up to December 2011 [3] and updated to January 15, 2014 [4], did not reach definitive conclusions about the potential risk of HIV acquisition associated with injectable progestins; the authors did not perform a meta-analysis because of concern about between-study heterogeneity, although this was not quantified statistically [3, 4]. A linked technical meeting of the World Health Organization in 2012 requested additional high-quality research to help better inform policy-makers, clinicians, and women about this important reproductive health issue [11].

Examining individual participant data (IPD) from several different studies can overcome some of the methodological limitations of reviews of aggregated data [12]. Our goal was to assess the risk of HIV acquisition associated with different hormonal contraceptives by combining data from large prospective longitudinal studies in an IPD meta-analysis. The specific objectives of this study were (1) to determine whether a woman’s hormonal contraceptive method increases the risk of HIV acquisition compared to women not using HC, (2) to evaluate whether age or herpes simplex virus type 2 (HSV-2) infection status modifies any effect of HC on the risk of HIV acquisition, and (3) to directly compare the risks of HIV acquisition between three groups of hormonal contraceptive (COC, DMPA, and NET-EN) users.

## Methods

The Protection of Human Subjects Committee of FHI 360 approved the study and judged it as exempt research (PHSC #10263). All included studies had relevant country-specific institutional ethical review and regulatory board approvals, and all participants within each study provided written informed consent for study participation.

The IPD meta-analysis followed a protocol (S1 Text) and a prespecified analysis plan (S2 Text). We report our findings in accordance with the Preferred Items of Reporting for Systematic Reviews and Meta-Analyses (PRISMA) (S1 Checklist) [13] and a checklist of items specific to IPD meta-analyses (S2 Checklist) [14].

### Study Eligibility and Inclusion Criteria

Cohort studies that prospectively collected data on both hormonal contraceptive use (COC, DMPA, or NET-EN) and incident HIV-1 infections in women aged 15 to 49 y from sub-Saharan Africa were eligible. We considered randomized controlled trials (RCTs) of HIV prevention interventions as cohort studies because HIV incidence is the outcome and many of these trials collected detailed longitudinal contraception data; several groups have published such secondary analyses of RCT data [15–20]. We excluded studies with <15 incident HIV infections, >5% missing HIV infection or HC data, or scheduled follow-up visits >6 mo apart.

### Information Sources

We used three sources of information. First, we used a database containing IPD from ten studies that contributed to an IPD meta-analysis of the effects of vaginal practices on the risk of HIV acquisition among women [21], amassed by the Vaginal Practices Research Partnership (VPRP). Second, we sought additional well-documented datasets from prospective cohort studies and RCTs completed by September 30, 2012, by asking collaborators and investigators of HIV prevention trials. Third, we checked the bibliographies of the two published systematic reviews for studies published up to December 2011 [1, 3]. We also checked the bibliography to January 15, 2014, of an updated version of one of the reviews [4].

### Study Selection

The ten studies contributing to the VPRP IPD meta-analysis [21] all had prospective hormonal contraceptive use data and met all other inclusion criteria. We reviewed the full text of all other identified publications and applied our inclusion and exclusion criteria. For eligible studies involving oral or vaginal microbicides that contained antiretroviral drugs, only data from the non-antiretroviral control arm were included (Table 1).

**Table 1:** Characteristics of studies included in the individual participant data meta-analysis.

Study Number and Country/Region [Reference]	Study Population	Primary Study Objective	Study Design	Participants Eligible for IPD Meta-Analysis	Planned Study Duration	Frequency of Follow-Up	Dates of Enrollment	Number of Participants	Mean (SD) Age at Enrollment(Years)	Median (IQR) Follow-Up (Months)	Percent Followed Up at 12 mo	Number of Incident HIV Infections	HIV Incidence, per 100 Woman-Years (95% CI)
1. Kenya [32, 39]	Women engaging in transactional sex	HC and HIV	Cohort	All	Not fixed	Monthly	02/93–12/02	1,219	27.1 (6.3)	11.6 (3.5–3.3)	65.3	162	11.8 (10.1–13.8)
2. South Africa [15, 41]	Women not previously screened for cervical cancer	Cervical cancer screening	RCT	Intervention (screening) and control	6–36 mo	6 mo	06/00–12/02	4,158	40.7 (4.2)	7.5 (5.8–12.2)	42.8	68	2.1 (1.6–2.7)
3. Uganda, Zimbabwe [30, 31]	Women attending RH clinics	HC and HIV	Cohort	All	15–24 mo	3 mo	11/99–09/02	4,425	25.4 (4.5)	23.2 (17.9–24.1)	97.2	211	2.8 (2.4–3.2)
4. Kenya [33]	Women engaging in transactional sex	HIV prevention; presumptive antibiotic treatment (azithromycin)	RCT	Intervention and control	24 mo	3 mo	05/98–01/02	399	28.8 (7.6)	22.7 (10.8–27.6)	76.8	30	4.6 (3.1–6.6)
5. Tanzania [34]	Women working in bars, guest houses	HIV prevention; microbicide feasibility study	Cohort	All	12 mo	3 mo	08/02–10/03	932	29.6 (7.8)	11.8 (8.5–12.0)	62.2	23	3.0 (1.9–4.5)
6. Tanzania [16, 40]	Women working in bars, guest houses	HIV prevention; HSV suppression (acyclovir)	RCT	Intervention and control	30 mo	3 mo	11/03–01/06	769	27.5 (5.0)	27.1 (15.5–28.3)	93.8	50	3.4 (2.5–4.5)
7. Zimbabwe, South Africa [20, 36]	Sexually active women	HIV prevention; diaphragm and condoms	RCT	Intervention and control	12–24 mo	3 mo	09/03–10/05	4,074	29.0 (7.8)	18.0 (14.8–23.9)	96.9	263	4.3 (3.8–4.9)
8. South Africa [57]	Women attending RH clinics	HC and HIV	Cohort	All	12 mo	3 mo	08/99–05/01	545	27.7 (6.6)	11.6 (10.8–12.0)	63.9	23	4.7 (3.0–7.1)
9. South Africa [37]	Women attending FP and postnatal clinics	HIV prevention; microbicide feasibility study	Cohort	All	12 mo	3 mo	01/02–01/04	690	24.7 (5.80)	11.1 (10.8–11.4)	38.8	20	3.4 (2.1–5.3)
10. South Africa [38]	Women attending FP clinics	HIV prevention; microbicide feasibility study	Cohort	All	12 mo	3 mo	07/03–07/04	257	29.0 (9.2)	9.5 (6.0–12.0)	56.8	29	15.2 (10.1–21.8)
11. Malawi, Zimbabwe [35, 42]	Women attending FP and postnatal clinics	HIV prevention; microbicide feasibility study	Cohort	All	9 mo	3 mo	06/01–08/02	1,423	28.0 (7.7)	9.0 (8.8–9.2)	82.1	52	5.2 (3.8–6.8)
12. South Africa [18, 43]	Sexually active women	HIV prevention; vaginal microbicide (Carraguard)	RCT	Intervention and control	9–24 mo	3 mo	03/04–06/06	5,615	29.8 (8.9)	17.7 (9.0–23.9)	92.6	272	3.7 (3.3–4.3)
13. Uganda [49]	Women engaging in transactional sex	HIV prevention; microbicide feasibility study	Cohort	All	12 mo	3 mo	04/08–05/09	418	26.5 (5.8)	12.0 (10.6–12.5)	65.5	17	4.4 (2.6–7.1)
14. Tanzania [48]	Women working in bars, guest house	HIV prevention; microbicide feasibility study	Cohort	All	12 mo	3 mo	07/08–09/09	873	27.9 (6.8)	11.4 (11.4–11.5)	94.1	30	3.9 (2.6–5.6)
15. East/southern Africa [17, 59]	Sexually active women	HIV prevention; HSV suppression (acyclovir)	RCT	Intervention and control	24 mo	3 mo	12/04–04/09	1,268	31.0 (7.7)	17.1 (11.9–23.7)	98.2	71	4.0 (3.1–5.0)
16. East/southern Africa [19, 44]	Sexually active women	HIV prevention; vaginal microbicide (PRO2000)	RCT	Intervention and control	12–24 mo	Monthly	10/05–08/08	8,596	29.3 (8.4)	12.0 (11.7–12.2)	94.8	413	4.4 (3.1–6.1)
17. South Africa [5]	Sexually active women	HIV prevention; vaginal antiretroviral (tenofovir)	RCT	Control only	Mean 18 mo	Monthly	05/07–01/09	444	23.5 (4.9)	19.1 (13.1–22.8)	99.1	60	9.1 (6.9–11.7)
18. East/southern Africa [6]	Sexually active women	HIV prevention; oral antiretroviral (Truvada)	RCT	Control only	Up to 60 wk	Monthly	06/09–04/11	1,019	24.2 (4.8)	10.2 (7.0–13.8)	91.2	36	4.4 (3.1–6.1)

FP, family planning; HSV, herpes simplex virus; IQR, interquartile range; RH, reproductive health; SD, standard deviation.

### Data Collection, Data Management, and Data Items

Two investigators (C.S.M. and P.C.) contacted the investigators of eligible component studies by email or phone to ask their permission to use their study data in the meta-analysis. We collected information for the individual studies included in this analysis from protocols, questionnaires, and publications, and asked study investigators to determine whether desired variables had been collected or could be derived. We used the existing structure of the VPRP database to include the data from additional studies, which included three levels of variables: study, individual, and visit level. Study-level variables consisted of country, study site, study design and population group(s), study aims, recruitment period, study duration, frequency of follow-up visits, planned follow-up duration for each woman, definitions of primary and secondary study outcomes, and diagnostic procedures. Individual-level variables included age, education, employment status, religion, socio-economic indicators, parity, and marital status. Visit-level variables were hormonal contraceptive use, pregnancy status, vaginal practices, numbers and type of sexual partners, coital frequency, transactional sex, condom use, sexual partner risk, and HIV, HSV-2, and other diagnosed sexually transmitted or reproductive tract infections. We included individual- and visit-level items for all study participants who had follow-up HIV and HC data. We excluded data from studies with scheduled follow-up visits >6 mo apart.

Three statisticians and data managers (P.C., C.K., and A. Bernholc) at the study coordinating center (FHI 360) worked closely with data managers and investigators of the individual studies to clarify issues about variable definition and missing, incomplete, or implausible data. Of the 18 included studies, datasets for 13 were provided either by their own data manager or through the VPRP. Investigators of the other five studies sent raw data to the coordinating center staff, who provided data management support.

### Primary Outcome and Exposure Measures

The primary outcome was incident HIV infection, defined as a new HIV infection following a preceding visit where the participant was confirmed HIV negative. The criteria for HIV diagnosis were defined by the investigators of the individual studies and were typically based on a positive ELISA/rapid test confirmed by a positive Western blot or HIV PCR test. The midpoint between the last negative and first positive HIV test was used as the estimated HIV infection date.

The primary exposure was hormonal contraceptive use with COCs (any preparation including estrogen plus progestin), DMPA (150 mg intramuscularly every 3 mo), or NET-EN (200 mg intramuscularly every 2 mo). COC, DMPA, and NET-EN use were recorded at each study visit. Studies that did not specify the type of injectable hormone were categorized as DMPA in the primary analysis because only South Africa had a significant number of NET-EN users. We examined in a sensitivity analysis the effect of limiting the meta-analysis to only studies where the injectable was specified. The comparison group was women not using hormonal contraceptives. This group included sterilized women, women using condoms (consistently or inconsistently), women using non-hormonal intrauterine devices or diaphragms, and women not using any modern contraceptive method.

Study participants were censored at the time they reported using a hormonal method not included in the study (such as the progestin-only pill or hormonal implants), at the end of the study, or at their last follow-up visit.

### Assessment of Study Methods and the Risk of Bias

We developed a list of methodological features of the component studies that could bias the estimates of the association between HC and HIV acquisition or affect their precision. We used criteria for items specific to the research question [3, 4, 22]. Additional criteria for cohort studies were drawn from the Newcastle-Ottawa Scale [23], the Downs and Black instrument [24], the Strengthening the Reporting of Observational Studies in Epidemiology Statement [25], and the Meta-Analysis of Observational Studies in Epidemiology checklist [26].

For each study we assessed documentation about the following items to classify the study as being at either lower or higher risk of bias: participant retention rate [3, 4, 22–27] (<80% versus ≥80%); measurement of important confounders (pregnancy, coital frequency, marital status/living with partner, and transactional sex [yes versus no]); measurement of contraceptive method use (every 3 mo or more frequently versus less frequently); and percentage of participants in a non-hormonal-contraceptive comparison group at baseline (≤10% versus >10%). Two investigators (C.S.M. and N.L.) independently evaluated each study and reached agreement about any differences through discussion. Studies for which all items were at lower risk of bias were categorized as “lower risk of bias,” and all other studies were categorized as “higher risk of bias,” for evaluating the association between HC and HIV.

### Statistical Analysis

We used Cox proportional hazards models with time-varying covariates to examine the association in each study between time-varying exposure to each hormonal contraceptive (COC, DMPA, and NET-EN) and HIV acquisition, and expressed the comparison with no hormonal contraceptive use as hazard ratios (HRs) with 95% confidence intervals. Follow-up time was censored at the first of the following: estimated date of HIV infection, the last follow-up visit, the end of the study, or after 30 mo of follow-up (owing to sparse data). The primary analysis used a two-stage approach to IPD meta-analysis; we used the effect estimate from each individual study and combined the effect estimates using random effects meta-analysis to estimate a summary HR (with 95% CI). We used the *I* ^2^ statistic to evaluate between-study heterogeneity (ranging from 0% to 100%) in this model and considered *I* ^2^ values below 50% as indicating mild to moderate heterogeneity [12, 28]. We examined the consistency of the results from the two-stage random effects model with those from a two-stage fixed effects model and with those from one-stage Cox regression analyses in which data were combined across all studies using study as the strata [28, 29]. No missing data were imputed in analyses; follow-up visits with a missing covariate did not contribute to the multivariable analyses. All statistical analyses were conducted using SAS (version 9.3, SAS Institute, Cary, North Carolina, US).

We constructed two multivariable models for each study: the primary analysis included a common set of covariates for each study (prespecified covariates were age, marital status/living with partner, condom use, and number of sex partners; region of study was added later to this group); the second model included specific covariates for each individual study that showed statistical evidence of confounding. We examined statistical evidence of confounding of the association between each hormonal contraceptive exposure and HIV infection in the individual studies. Each potential confounding factor was added to a model that included hormonal contraceptive exposure and the prespecified covariates. If addition of the variable resulted in the HR changing by ≥10% for any of the hormonal contraceptive exposures, we included the covariate in the multivariable model. Variables evaluated for confounding included region of study, recent sexual behavior (concurrent sex partners, coital frequency, transactional sex, anal sex, oral sex), vaginal practices, reproductive health factors (parity, pregnancy history and status, lactation status), physical exam variables (cervical ectopy, genital epithelial findings), presence of cervical infections (*Chlamydia trachomatis*, *Neisseria gonorrhoeae*), and presence of vaginal infections (bacterial vaginosis, *Trichomonas vaginalis*, vulvovaginal candidiasis).

We examined statistical evidence for effect modification using likelihood ratio tests. If the *p*-value was <0.05, we present the stratified results. Prespecified study objectives were to determine whether associations between use of each hormonal contraceptive method (COC, DMPA, and NET-EN) and HIV acquisition differed among young women (15–24 y) and older women (25–49 y) and whether HSV-2 infection status altered the effect of HC on the risk of HIV acquisition [30, 31]. We conducted the following prespecified subgroup analyses: risk of methodological bias in component studies (higher risk versus lower risk of bias), region of study (southern Africa versus South Africa versus east Africa), and underlying HIV incidence (higher versus lower in the non-hormonal-contraceptive comparison group for each study). We also did prespecified sensitivity analyses to examine hormonal contraceptive method switching (censoring at last visit before switching), limiting the analyses to only studies where the type of injectable was specified, accounting for pregnancy status (two methods: excluding all women who became pregnant during the study and censoring women at the last visit prior to pregnancy), and limiting person-time to periods with no condom use (by including only person-time from women never reporting condom use and person-time up to the time in a study that a woman first reported condom use). In post hoc subgroup analyses we examined whether engaging in transactional sex modified the relationship between HC and HIV infection and examined results separately for cohort studies and RCTs. We also explored whether our findings would differ if we added the results of eligible studies for which we could not obtain individual-level data.

## Results

**Fig 1:**
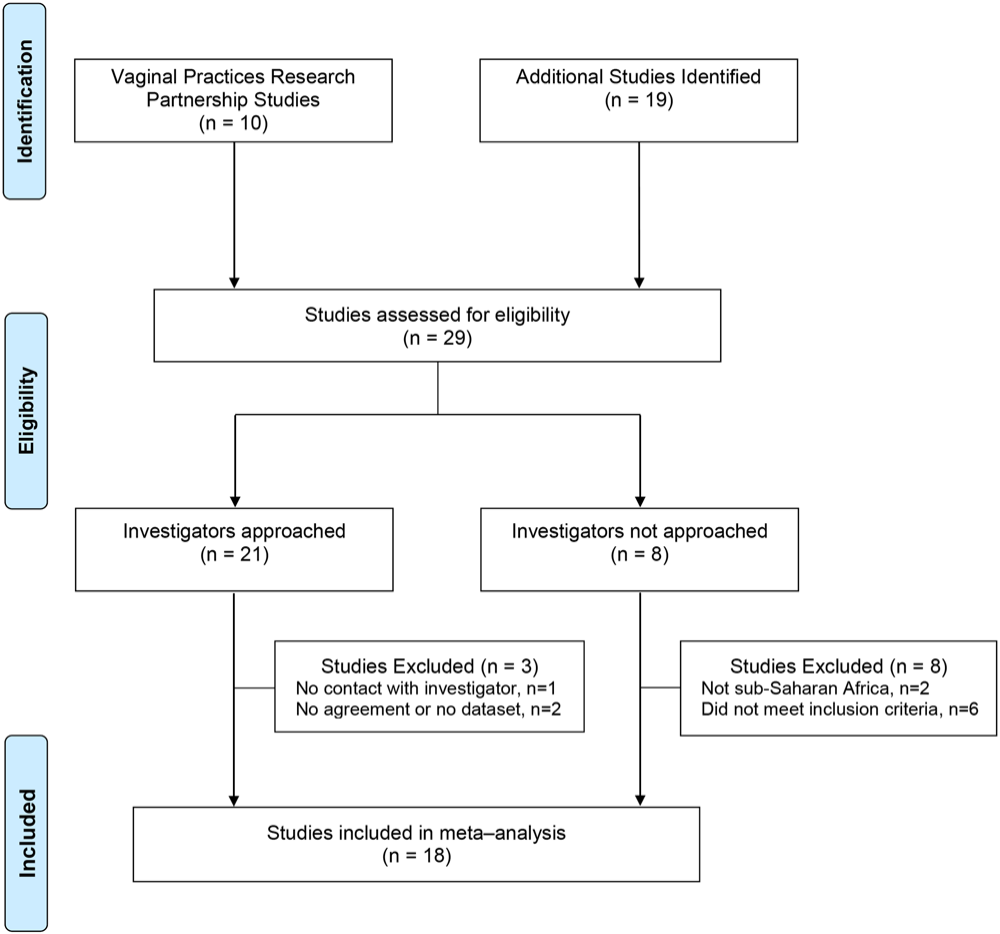
Flow diagram of studies included in individual participant data meta-analysis of hormonal contraception and HIV acquisition.

We included the ten studies [15, 16, 20, 30, 32–42] (Table 1) that contributed to the VPRP meta-analysis [21]. Our search strategy identified 19 additional potentially eligible studies (Fig. 1) [6, 43–59]. We excluded 11 of these studies: two were not conducted in sub-Saharan Africa [51, 56], and six did not meet the other inclusion criteria because they had >5% missing exposure data [54], follow-up visits >6 mo apart [52, 53, 55], or no longitudinal data (from visits ≤6 mo apart) on injectable contraception [5, 50]. For two studies we did not reach an agreement with the study investigators to use their datasets by our cutoff date of September 2012 [47, 58]; we could not make contact with the responsible investigator of one study despite repeated attempts [45]. Additional searches after September 2012 identified one additional published study that did not meet the inclusion criteria [60] and two conference abstracts from studies that fulfilled the inclusion criteria [61, 62] (Table 2).

**Table 2:** Characteristics of studies not included in the individual participant data meta-analysis.

**Study Number and Country/Region**	**Short Study Name**	**Primary Publications [Reference]**	**Reason Not Included in IPD Meta-Analysis**	**Study Design**	**Number of Women with HIV/Total Number of Women**	**Number of Women with HIV in Contraceptive Groups**	**Comparison Group^a^**	**Study Results: Adjusted Effect Measure (95% CI) unless Otherwise Specified^b^**
**Studies that did not meet inclusion criteria (*n* = 8)**								
19. Rwanda	Not known	Bulterys et al., *AIDS* 1994 [55]	Did not meet inclusion criteria: follow-up visits >6 mo apart	Cohort	31/1,524	12	No contraception	HC use: OR 1.9 (0.8–4.6)
20. Thailand	Not known	Ungchusak et al., *JAIDS* 1996 [51]	Not sub-Saharan Africa	Cohort	15/365	NR	No contraception	COC: IRR 0.22 (0.03–1.9); injectable HC: IRR 3.8 (1.0–14.4)
21. Tanzania	Not known	Kapiga et al., *AIDS*1998 [53]	Did not meet inclusion criteria: follow-up visits >6 mo apart	Cohort	75/2,471	7 (COC); 2 (injectable HC)	No COC use; no injectable use	COC: HR 1.0 (0.5–2.3); injectable HC: HR 0.3 (0.07–1.3)
22. Thailand	Not known	Kilmarx et al., *AIDS* 1998 [56]	Not sub-Saharan Africa	Cohort	30/340	20 (COC); 5 (DMPA)	No COC use; no DMPA use	COC: RR 1.8 (0.8–4.0); DMPA: unadjusted RR 1.5 (0.6–4.0)
23. Uganda	Rakai Study	Kiddugavu et al., *AIDS* 2003 [52]	Did not meet inclusion criteria: follow-up visits >6 mo apart	Cohort	202/5,117	12 (COC); 16 (injectable HC)	No contraception, no condoms	COC: IRR 1.1 (0.5–2.6); injectable HC: IRR 0.8 (0.4–1.7)
24. Benin, Ghana, Nigeria, South Africa, Uganda, India	SAVVY/CS Trials	Feldblum et al., *Sex Transm Dis* 2010 [50]	Did not meet inclusion criteria: no longitudinal data on injectable contraception	RCT	114/7,364	13 (COC); 15 (injectable HC)	No contraception or emergency contraception only	COC: unadjusted RR 1.8 (0.8–4.1); injectable HC: unadjusted RR 2.5 (1.1–5.6)
25. South Africa	CAPRISA 050/051	Abdool Karim et al., *Int J Epidemiol* 2011 [46]	Did not meet inclusion criteria: longitudinal data on injectable contraception >6 mo apart	RCT	39/594	NA	NA	No published HC—HIV results
26. South Africa, Zambia, Zimbabwe	HPTN 039	Reid et al., *JAIDS* 2010 [54]	Did not meet inclusion criteria: >5% missing data for exposure	RCT	72/1,358	NR	No contraception, no condoms	COC: HR 0.9 (0.5–1.8); injectable HC: HR 0.9 (0.5–1.9)
**Studies not included because agreement was not obtained from investigators (*n* = 3)**								
27. South Africa, west Africa, Southeast Asia	COL 1492	Van Damme et al., *Lancet* 2002 [45]	No agreement/dataset	RCT	99/552	NA	NA	No published HC—HIV results
28. Malawi, South Africa, Zambia, Zimbabwe, US	HPTN 035	Abdool Karim et al., *AIDS* 2011 [58];; Chirenje et al., Int’l Microbicides Conf. 2012 [61]	No agreement/dataset	RCT	192/2,887	NR	No HC	COC: HR 0.6 (0.3–1.2); injectable HC: HR 1.4 (0.9–2.0)
29. Kenya, Uganda	Partners Prep	Baeten et al., *N Engl J Med* 2012 [47]	No agreement/dataset	RCT	28/1,584	NA	NA	No published HC—HIV results
**Studies with results published after September 2012 (*n* = 2)**								
30. Uganda	Rakai Study	Lutalo et al., *AIDS* 2013 [60]	Does not meet inclusion criteria: follow-up visits >6 mo apart; published after meta-analysis dataset closed	Cohort	30/190	3 (COC); 7 (DMPA)	No contraception, no condoms	COC: IRR 2.7 (0.82–8.6); DMPA: IRR 1.4 (0.6–3.4)
31. South Africa, Uganda, Zimbabwe	VOICE Study	Noguchi et al., CROI 2014 [62]	Published after meta-analysis dataset closed; no non-hormonal-contraceptive comparison	RCT	207/3,141	204	NET-EN use	DMPA: HR 1.4 (1.0–2.0)

^a^Comparison group, based on information reported by the authors; for study 31 there was no comparison with non-hormonal-contraceptive users, so the comparison group is women using NET-EN.

^b^“Injectable” reported if this was the term used by the authors and the specific progestin was not mentioned; all effect sizes rounded to one decimal place.

IRR, incidence rate ratio; NA, not applicable; NR, not reported; OR, odds ratio; RR, risk ratio.

### Description of Studies and Study Populations

Overall, there were nine cohort studies [30, 32, 34, 35, 37, 38, 48, 49, 57] and nine RCTs [5, 6, 15, 16, 17, 33, 36, 43, 44] (Table 1; S1 Table). The 18 studies were conducted in nine countries. Of the 37,124 participants, 27% were from east Africa (Kenya, Uganda, Tanzania, Rwanda), 55% were from South Africa, and 18% were from other southern African countries (Zambia, Zimbabwe, Malawi, Botswana). Participants were sexually active women recruited from community settings, reproductive health or family planning clinics, or bars and other recreational facilities where high levels of HIV infection have been documented (Table 1). Most studies followed women for 12 to 24 mo with clinic visits monthly, quarterly, or every 6 mo. Retention at 12 mo ranged from 39% to 99%. Studies documented from 17 [49] to 413 [44] incident HIV infections, with infection rates ranging from 2.1 [15] to 15.2 [38] per 100 woman-years; most studies recorded incidence rates between 2.5 and 5.0 per 100 woman-years.

Five of the 18 included studies were judged to be at lower risk of bias for the analysis of HC and HIV acquisition [17, 18, 20, 30, 48] (Table 3). Amongst the other 13 studies, eight had <80% retention at 12 mo, eight did not include a minimal set of confounders judged to be important, one did not measure HC every 3 mo or more often, and two had <10% of study participants in a no-HC comparison group.

**Table 3:** Assessment of risk of bias for the 18 studies included in the individual participant data meta-analysis.

**Study Number and Country/Region**	**≥ 80% Retention Rate**	**Measurement of Important Confounders^a^**	**Contraceptive Method Measured Every 3 mo or More Frequently**	**≥ 10% in No-HC Comparison Group**	**Lower Risk of Bias^b^**
1. Kenya [32, 39]	No	Yes	Yes	Yes	No
2. South Africa [15, 41]	No	No	No^c^	Yes	No
3. Uganda, Zimbabwe [30, 31]	Yes	Yes	Yes	Yes	Yes
4. Kenya [33]	No	No	Yes	Yes	No
5. Tanzania [34]	No	Yes	Yes	Yes	No
6. Tanzania [16, 40]	Yes	No	Yes	Yes	No
7. Zimbabwe, South Africa [20, 36]	Yes	Yes	Yes	Yes	Yes
8. South Africa [57]	No	No	Yes	Yes	No
9. South Africa [37]	No	No	Yes	Yes	No
10. South Africa [38]	No	No	Yes	Yes	No
11. Malawi, Zimbabwe [35, 42]	Yes	No	Yes	Yes	No
12. South Africa [18, 43]	Yes	Yes	Yes	Yes	Yes
13. Uganda [49]	No	Yes	Yes	Yes	No
14. Tanzania [48]	Yes	Yes	Yes	Yes	Yes
15. East/southern Africa [17, 59]	Yes	Yes	Yes	Yes	Yes
16. East/southern Africa [19, 44]	Yes	No	Yes	Yes	No
17. South Africa [5]	Yes	Yes	Yes	No	No
18. East/southern Africa [6]	Yes	Yes	Yes	No	No

^a^Important confounders include pregnancy status, coital frequency, marital status/living with partner, transactional sex; “yes” indicates all measured; “no” indicates one or more missing.

^b^Lower risk of bias based on “yes” to all: ≥80% followed at 12 mo, measurement of important confounders, contraceptive method measured every 3 mo or more frequently, and ≥10% in no-HC comparison group.

^c^Follow-up visits every 6 mo.

### Contraceptive Use

At baseline about half of the women (18,216/36,973) were not using HC (the comparison group), 26% (9,722/36,973) were using DMPA, 16% (5,835/36,973) were using COCs, and 9% (3,200/36,973) were using NET-EN. Although DMPA was used by about a quarter of women in all three regions, COC use was lower in South Africa (1,721/20,449, 8%) than in other southern African countries (2,578/6,645, 39%). Most NET-EN use was in South Africa, and the highest proportion of women not using HC was in east Africa (5,778/9,859, 59%). During follow-up, 67% (23,628/35,090) of women remained on the same contraceptive method. Eight percent (1,424/18,464) of women originally using a hormonal contraceptive method switched to another hormonal method, while 14% (2,630/18,464) discontinued HC. Among those not using HC at baseline, 17% (2,783/16,626) initiated a hormonal method during follow-up. Thirteen percent (4,625/35,090) of women overall switched contraceptive methods multiple times.

### Contraceptive Use and HIV Acquisition

Across studies there were 1,830 incident HIV infections, for an overall incidence of 4.2 per 100 woman-years. Based on time-varying exposure to contraceptive method, HIV incidence was highest among DMPA users (5.1 per 100 woman-years), followed by NET-EN users (4.8 per 100 woman-years), the no-HC group (3.9 per 100 woman-years), and COC users (3.4 per 100 woman-years).

**Fig 2:**
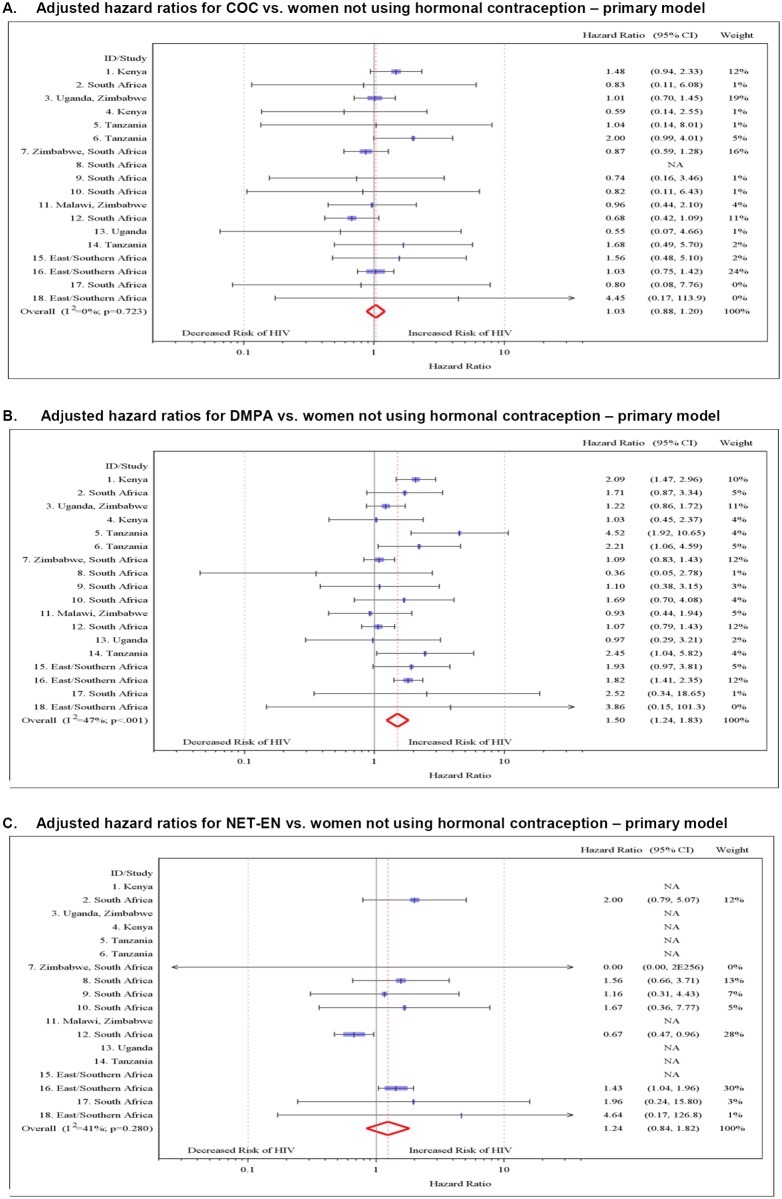
Multivariable associations between hormonal contraceptive use and HIV acquisition by study, with non-hormonal-contraceptive group as the reference. (A) aHRs for COC use versus no HC use—primary model. (B) aHRs for DMPA use versus no HC—primary model. (C) aHRs for NET-EN use versus no HC—primary model. Individual study results from Cox regression modeling. Pooled aHRs from random effects meta-analysis adjusted for age, married/living with partner, number of sex partners, condom use, and region (east Africa, southern Africa, and South Africa). Each horizontal line represents the 95% confidence interval around the HR. Shaded areas represent the comparative weight of each study. No estimate was possible if there were not events in the specified contraceptive group. NA, not applicable.

In univariable analyses, COC use was not associated with HIV acquisition (HR 1.01, 95% CI 0.84–1.21), while DMPA use (HR 1.56, 95% CI 1.31–1.86) and NET-EN use (HR 1.51, 95% CI 1.21–1.98) were. In multivariable analyses using a two-stage random effects approach and controlling for a common set of covariates for each study (region plus the prespecified covariates age, marital status/living with partner, number of sex partners, and condom use), we found no association between COC use and HIV acquisition (adjusted HR [aHR] 1.03, 95% CI 0.88–1.20) (Table 4; Fig. 2), DMPA was associated with an increased risk of HIV acquisition (aHR 1.50, 95% CI 1.24–1.83), and the association between NET-EN use and HIV acquisition became weaker (aHR 1.24, 95% CI 0.84–1.82). Between-study heterogeneity was mild for each analysis (*I* ^2^ = 0% for COC, *I* ^2^ = 47% for DMPA, and *I* ^2^ = 41% for NET-EN). Results from the two-stage fixed effects and the one-stage meta-analysis models were very similar to those of the two-stage random effects model (S2 Table).

**Table 4:** Hormonal contraception—HIV individual participant data meta-analysis: hazard ratios for sensitivity and subgroup analyses.

**Analyses**	**Number of HIV Infections/Woman- Years**	**Time-Varying COC^a^**		**Time-Varying DMPA^a^**		**Time-Varying NET-EN^a^**	
		**HR (95% CI)^b^ or *p*-Value**	**aHR (95% CI)^b^**	**HR (95% CI)^b^ or *p*-Value**	**aHR (95% CI)^b^**	**HR (95% CI)^b^ or *p*-Value**	**aHR (95% CI)^b^**
**Overall results**	1,743/42,041	1.01 (0.84–1.21)	1.03 (0.88–1.20)	1.56 (1.31–1.86)	1.50 (1.24–1.83)	1.51 (1.21–1.90)	1.24 (0.84–1.82)
**Age**							
15–24 y of age	858/15,194	0.89 (0.65–1.23)	0.91 (0.72–1.16)	1.33 (1.04–1.71)	1.25 (1.00–1.58)	1.08 (0.81–1.44)	0.96 (0.72–1.29)
25+ y of age	885/26,847	1.10 (0.85–1.43)	1.17 (0.88–1.56)	1.70 (1.27–2.29)	1.69 (1.25–2.28)*	1.54 (0.71–3.34)	1.38 (0.63–3.04)*
Age by HC interaction		*p =* 0.52		*p =* 0.38		*p =* 0.87	
**HSV-2 status at baseline**							
HSV-2 negative	317/10,597	1.11 (0.56–2.18)	1.23 (0.69–2.21)*	1.47 (1.11–1.93)	1.61 (1.09–2.36)	1.49 (0.92–2.42)	1.14 (0.69–1.88)
HSV-2 positive	957/19,400	1.05 (0.81–1.36)	1.10 (0.87–1.39)	1.71 (1.28–2.28)	1.60 (1.18–2.17)*	2.19 (1.52–3.15)	1.61 (1.09–2.37)
HSV-2 by HC interaction		*p =* 0.60		*p =* 0.70		*p =* 0.31	
**Risk of bias assessment**							
Higher risk of bias	908/11,957	1.18 (0.95–1.46)	1.16 (0.93–1.45)	1.77 (1.42–2.20)	1.73 (1.39–2.16)	1.88 (1.46–2.43)	1.50 (1.14–1.96)
Lower risk of bias	835/30,084	0.87 (0.64–1.19)	0.91 (0.73–1.14)	1.29 (1.10–1.52)	1.22 (0.99–1.50)	1.04 (0.74–1.45)	0.67 (0.47–0.96)
Risk of bias by HC interaction		*p =* 0.13		*p <*0.001		*p <*0.001	
**Pregnancy**							
Time-varying pregnancy	1,580/39,059	1.02 (0.83–1.24)	1.01 (0.85–1.19)	1.60 (1.34–1.91)	1.48 (1.18–1.85)*	1.51 (1.21–1.90)	1.32 (0.79–2.19)*
Censoring pregnant visits	1,522/38,609	0.99 (0.77–1.28)	0.99 (0.83–1.17)	1.65 (1.33–2.04)	1.58 (1.25–2.01)*	1.6 (1.22–2.10)	1.29 (0.84–1.99)
Excluding women ever pregnant	1,522/36,057	1.01 (0.78–1.30)	1.00 (0.84–1.19)	1.59 (1.27–1.99)	1.51 (1.18–1.93)*	1.57 (1.27–1.94)	1.25 (0.83–1.89)
**No HC switch^c^**	1,178/31,450	0.96 (0.74–1.24)	1.01 (0.81–1.25)	1.55 (1.25–1.93)	1.51 (1.20–1.89)	1.65 (1.28–2.12)	1.28 (0.82–1.99)
**Studies with specified injectable HC**	1,446/34,510	1.11 (0.94–1.32)	1.07 (0.90–1.28)	1.67 (1.37–2.05)	1.61 (1.28–2.03)	1.55 (1.14–2.11)	1.28 (0.82–2.00)
**No condom use^d^**	332/12,535	1.29 (0.86–1.94)	1.08 (0.71–1.65)	1.62 (1.22–2.15)	1.6 (1.11–2.31)	1.55 (0.93–2.59)	1.32 (0.65–2.69)
**Region of study**							
East Africa	483/13,085	1.49 (1.14–1.96)	1.58 (1.19–2.09)	2.05 (1.66–2.54)	2.09 (1.68–2.60)	NA	NA
Southern Africa	324/8,506	0.77 (0.58–1.00)	0.81 (0.61–1.06)	0.91 (0.68–1.20)	0.94 (0.70–1.26)	1.06 (0.30–3.74)	1.18 (0.33–4.25)
South Africa	936/20,450	0.89 (0.69–1.16)	0.83 (0.63–1.08)	1.46 (1.25–1.70)	1.30 (1.11–1.53)	1.38 (1.11–1.70)	1.17 (0.81–1.70)
Region by HC interaction		*p =* 0.004		*p <*0.001		*p =* 0.94	
**HIV incidence^e^**							
Population with low HIV incidence	1,043/30,083	1.07 (0.88–1.29)	1.01 (0.82–1.25)	1.67 (1.32–2.11)	1.64 (1.23–2.19)*	1.48 (0.98–2.24)	1.15 (0.66–1.99)*
Population with high HIV incidence	625/11,957	0.91 (0.59–1.39)	1.06 (0.81–1.37)	1.4 (1.04–1.88)	1.34 (0.98–1.83)	1.75 (0.83–3.67)	1.59 (0.75–3.38)
HIV incidence by HC interaction		*p =* 0.36		*p =* 0.122		*p =* 0.35	
**Transactional sex**							
Population with transactional sex	319/5,427	1.46 (1.06–2.02)	1.51 (1.09–2.10)	1.86 (1.44–2.39)	1.76 (1.29–2.40)	1.71 (0.09–33.99)	0.92 (0.06–13.43)
Population with no transactional sex	1,424/36,614	0.92 (0.75–1.15)	0.96 (0.80–1.15)	1.50 (1.23–1.81)	1.45 (1.16–1.80)	1.52 (1.20–1.92)	1.25 (0.84–1.85)
Transactional sex by HC interaction		*p =* 0.03		*p =* 0.09		*p =* 0.70	

^a^“Time-varying” signifies that the contraceptive variable may vary with time, i.e., vary at each visit for a particular participant. Each HC group is compared to women not using HC.

^b^Adjusted for region, age, married/living with partner, time-varying >1 sex partner, time-varying condom use.

^c^Data censored at last visit prior to first contraceptive method switch.

^d^Data censored at last visit prior to first reported condom use.

^e^Incidence measured low versus high (based on median: <3.9 versus ≥3.9 per 100 woman-years) in the no-HC comparison group.

* *I* ^2^ value 50%–75%; otherwise all *I* ^2^ values are <50%.

NA, not applicable.

In multivariable models that included specific covariates for each study, we found associations with HIV acquisition similar to those for the primary model that controlled for the same covariates in all studies: the aHR for COC use was 1.07 (95% CI 0.91–1.25), for DMPA use was 1.52 (95% CI 1.27–1.82), and for NET-EN use was 1.27 (95% CI 0.99–1.61) (S2 Table).

**Fig 3:**
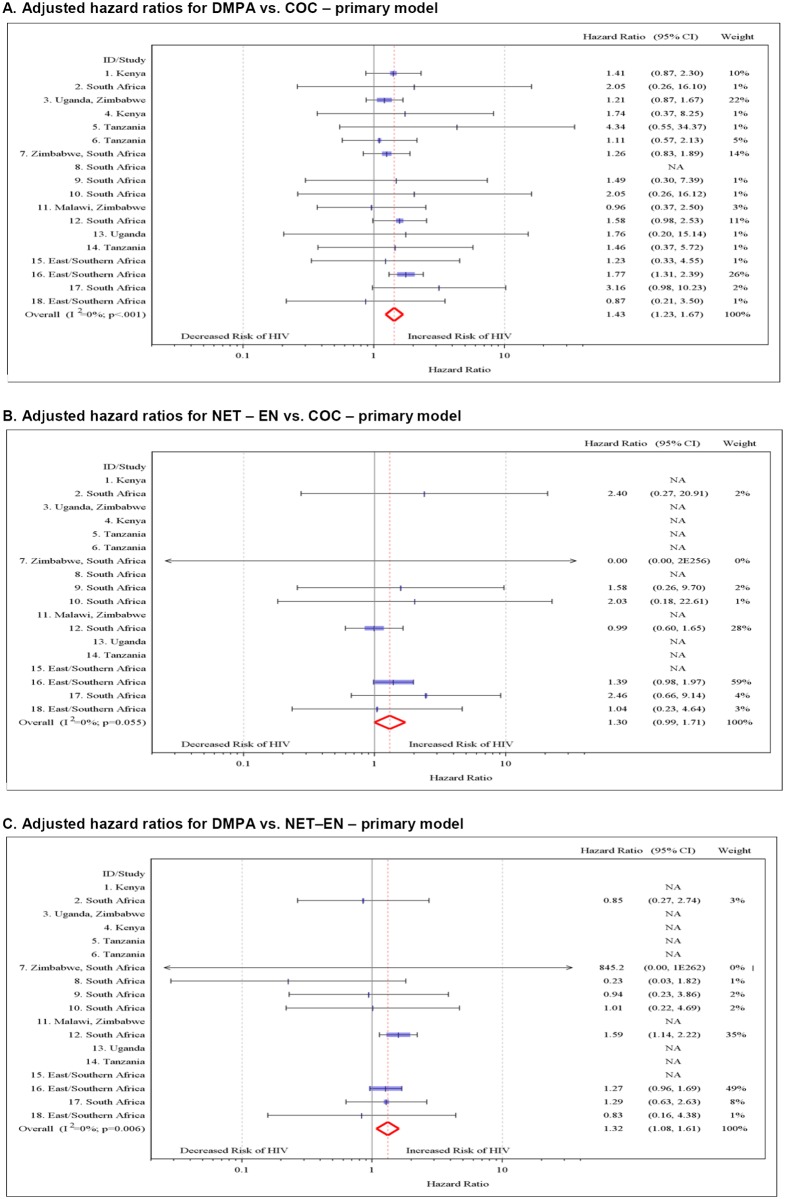
Multivariable associations directly comparing different hormonal contraceptives and HIV acquisition by study. (A) aHRs for DMPA versus COC use—primary model. (B) aHRs for NET-EN versus COC use—primary model. (C) aHRs for DMPA versus NET-EN use—primary model. Individual study results from Cox regression modeling. Pooled aHRs from random effects meta-analysis adjusted for age, married/living with partner, number of sex partners, condom use, and region (east Africa, southern Africa, and South Africa). Each horizontal line represents the 95% confidence interval around the HR. Shaded areas represent the comparative weight of each study. No estimate was possible if there were not events in the specified contraceptive group.

In direct comparisons between the three hormonal methods, we found that DMPA use was associated with an increased risk of HIV acquisition compared with both COC use (aHR 1.43, 95% CI 1.23–1.67) and NET-EN use (aHR 1.32, 95% CI 1.08–1.61) (Fig. 3). There was also some evidence of increased risk for NET-EN use compared with COC use (aHR 1.30, 95% CI 0.99–1.71, *p* = 0.055).

### Interactions between Hormonal Contraception and Age and HSV-2 Status

We found no statistical evidence for an interaction between age or HSV-2 infection status and HC on HIV acquisition (Table 4).

### Sensitivity Analyses

We found stronger associations between hormonal contraceptive use and HIV acquisition in the group of studies at higher risk of bias than in those at lower risk of bias (Table 4). Statistical evidence of interaction was stronger for DMPA use (*p*_interaction_ = 0.002) and NET-EN use (*p*_interaction_ = 0.005) than for COC use (*p*_interaction_ = 0.133). In the group of studies at lower risk of bias, the aHR compared to no HC use was 0.91 (95% CI 0.73–1.14) for COC use, 1.22 (95% CI 0.99–1.50) for DMPA use, and 0.67 (95% CI 0.47–0.96) for NET-EN use. In post hoc analyses of individual items associated with risk of bias, there was evidence of stronger associations in studies with lower retention rates (<80%) than in studies with higher retention rates (≥80%) for DMPA use (*p*_interaction_ = 0.094) and NET-EN use (*p*_interaction_ = 0.035) but not for COC use (*p*_interaction_ = 0.235), and in studies missing important confounding variables for NET-EN use (*p*_interaction_ = 0.015) but not in studies missing important confounding variables for DMPA use (*p*_interaction_ = 0.225) or COC use (*p*_interaction_ = 0.502) (S2 Table). Only one study had an interval between hormonal contraceptive measurements of >3 mo. There was no statistical evidence of an interaction between HC and a study having less than 10% of its sample in the no-HC group.

Sensitivity analyses where we censored visits at the time a woman became pregnant, and an analysis where women who ever became pregnant during the study were excluded, resulted in estimates of effects for each of the contraceptive methods that were very similar to the primary study results (S2 Table).

We found stronger associations between hormonal contraceptive use and HIV acquisition in east Africa than in southern Africa or South Africa for COC use (*p*_interaction_ = 0.004) and DMPA use (*p*_interaction_ < 0.001). Interactions between region of study and NET-EN were not meaningful because NET-EN was used primarily in South Africa (Table 4). There was increased risk associated with DMPA use for east Africa and South Africa but not for southern Africa, and an increased risk for COC use in east Africa but not in South Africa or Southern Africa.

We found no statistical evidence for modification of the association between HC and HIV acquisition according to the background HIV incidence of the component studies. Prespecified sensitivity analyses using different methods for censoring person-time prior to contraceptive method switch, limiting studies to only those where the type of injectable was specified, or limiting person-time to periods with no condom use yielded results very similar to the primary study results (Table 4). In post hoc analyses, we found some evidence for stronger associations between COC (*p*_interaction_ = 0.025) and DMPA (*p*_interaction_ = 0.088) use and HIV acquisition in women reporting transactional sex than among women not reporting transactional sex (Table 4). We found no important differences between analyses of HC and HIV acquisition based on type of study design (RCT or cohort study) or after including the published results from the one study for which we could not obtain individual-level data [61] (S2 Table).

## Discussion

In this large IPD meta-analysis, we found that women who use DMPA had an increased risk of HIV acquisition compared to women not using HC, after controlling for potential confounding variables. The incidence of HIV was also increased for women using NET-EN, but confidence intervals were wide, and the increase was not statistically significant after controlling for potential confounding factors. There was no increased HIV risk associated with COC use. However, the assessed risk of methodological bias of component studies modified the effect of the hormonal contraceptive methods on HIV acquisition, with lower HRs for all contraceptive methods in studies at lower risk of bias. Direct comparisons between the three contraceptives suggest that use of DMPA is associated with an increased risk of HIV acquisition compared to either COC or NET-EN use and that NET-EN use is associated with a borderline increase in HIV acquisition risk compared to women using COCs (*p* = 0.055). Neither age nor baseline HSV-2 infection status modified the effect of the hormonal contraceptive methods on HIV acquisition. The findings from other sensitivity analyses support the overall study findings.

Our finding that oral contraceptive use is not associated with increased risk of HIV, compared with no hormonal contraceptive use, is consistent with descriptive reviews of the findings from most previous prospective studies [3, 4]. Overall, we found a 50% higher risk of HIV acquisition in women using DMPA, but the increase was reduced to 22% in studies at lower methodological risk of bias, with confidence intervals including the possibility of no increased risk. Our meta-analysis results concerning DMPA agree with the findings of an increased HIV risk in some studies [17, 31, 35, 50, 51, 63], but do not agree with the findings of other studies [15, 16, 18, 52–56]. Our meta-analysis is, to our knowledge, the largest analysis to date of the association between NET-EN and HIV acquisition. Our findings indicate no overall increased HIV risk associated with NET-EN use; in studies at lower risk of bias, the risk of HIV was even lower. This is in agreement with five prospective studies in NET-EN users [15, 18–20, 57]. Our finding of an increased risk of HIV acquisition associated with DMPA use when directly compared with NET-EN use agrees closely with a secondary analysis of data from the VOICE (Vaginal and Oral Interventions to Control the Epidemic) microbicide trial (DMPA versus NET-EN aHR 1.44, 95% CI 1.05–1.98; Table 2) [62], which ended after data collection in our study was completed.

The quantitative results from this IPD meta-analysis provide several advances over previous reviews of HC and HIV [1, 3, 4]. First, we provide pooled summaries of associations between injectable progestin-only contraceptives and HIV acquisition. Second, the individual-level data allowed a consistent approach to coding and multivariable analysis [14, 64], which overcame some of the heterogeneity that precluded meta-analysis of the aggregated data [3, 4]. Third, with data from about 37,000 women and more than 1,800 incident HIV outcomes, we had sufficient statistical power to examine associations between specific contraceptives and HIV risk and to investigate effect modification in prespecified subgroup analyses. In particular, we found that methodological features of study design or conduct affected the association between hormonal contraceptive use and HIV acquisition. Assessing the risk of bias in observational studies is inherently subjective, so we tried to minimize the subjectivity of these ratings by having independent evaluations by two evaluators. We developed our own assessment tool a priori and included items from published checklists together with items related to other methodological features we believed to be important for studies of HC and HIV risk. Other evaluators have chosen different criteria [3, 4].

It is biologically plausible that DMPA might be more strongly associated with an increased risk of HIV acquisition than either NET-EN or COCs. Two particular characteristics of DMPA are worth noting. First, DMPA results in a more hypoestrogenic environment than NET-EN and estrogen-containing COCs [65, 66], and lack of estrogen has been linked to increased HIV risk through decreased integrity of the vaginal epithelium and changes to the genital immune environment [10, 67]. Second, medroxyprogesterone acetate has a higher affinity for binding with the glucocorticoid receptor than either norethindrone or levonorgestrel (progestins used in NET-EN and most COCs in this study), and activation of the glucocorticoid receptor has been linked to suppressed local immunity in several studies [68–70]. Other factors associated with hormonal contraceptive use might also increase the risk of HIV acquisition, such as changes in the genital epithelium (e.g., cervical ectopy), changes in the vaginal microbiome [71–73], changes in the genital immune environment [74–76], and direct effects on HIV (i.e., up-regulation of viral replication) [1, 10, 70, 75].

Our meta-analysis has some limitations. First, our collection of datasets might not be representative of all datasets that could be used to address the associations between HC and HIV. This problem affects reviews of observational epidemiology in general because many studies are secondary analyses of existing datasets; in other studies, the associations of interest may not have been analyzed or published. Systematic searches of electronic databases will only identify the published studies. Our search strategy identified both published studies and datasets with the relevant variables that had not yet been analyzed. The 18 datasets in our meta-analysis included the three studies specifically designed to investigate the research question [30, 32, 57], most of the studies included in a systematic review [3, 4], and ten new datasets [5, 6, 16, 19, 33–35, 37, 38, 49]. Reasons for excluding studies were independent of the study findings. Our findings did not change with the addition of the one eligible study with HC—HIV results from among the datasets we were not able to obtain [61] (S2 Table). Second, while IPD meta-analysis overcomes some of the problems associated with aggregated data, it cannot eliminate bias stemming from study design or conduct. In particular, not all component studies had comparable data on all subgroups and potential confounding variables. We worked directly with the primary investigators from all included studies to try to define variables consistently, but residual confounding could still be present in our effect estimates. Third, the studies in the meta-analysis used self-reported measures of sexual behavior, including condom use, which might not be accurate. If over-reporting of condom use is primarily in the HC groups (compared to the no-HC group), then the effect of this misreporting would likely be to overinflate the effect estimates for HC. Conversely, if over-reporting of condom use is greater in the no-HC group than in the HC groups (as we believe is more likely, given the higher self-reported condom use in the no-HC group than in the HC groups), then such over-reporting will result in an underestimate of the true effect of hormonal contraceptives on HIV acquisition. In any case, we included a sensitivity analysis where person-time was limited to those periods when women reported no condom use, and there was little change in the effect measures for any of the hormonal contraceptives. The reports of no condom use are thought to be of higher validity, as there is little social pressure to underreport condom use in these studies. Fourth, there was evidence of between-study heterogeneity in the main analyses for NET-EN and DMPA, albeit mild. The *I* ^2^ values for DMPA (*I* ^2^ = 47%) and NET-EN (*I* ^2^ = 41%) were similar, but the patterns of results differed. In the forest plot of NET-EN and HIV, there was one statistically influential study with an effect estimate in the opposite direction from the other studies (Fig. 2C, study #12). We evaluated this pattern and found that this study was not statistically an outlier [77]. Finally, marginal structural Cox survival models using stabilized inverse probability treatment weighting might have been a more appropriate approach to control time-dependent confounding than Cox proportional hazards models [3, 78, 79]. However, we could not apply this method consistently across the different studies.

This IPD meta-analysis found no evidence that COC or NET-EN use increases women’s risk of HIV compared to women not using HC, and adds to the evidence that DMPA might increase the risk of HIV acquisition, although some of the excess risk attributed to injectable contraception results from methodological limitations of the studies, including poor follow-up and residual confounding. Because of the importance of effective family planning to women’s reproductive health and to the morbidity and mortality of women and children, it is critical to obtain the highest quality evidence possible to inform the decisions of women, clinicians, and policy-makers in regions or risk groups with high HIV incidence. The results of this study also provide important information to inform the design of an RCT [80, 81], which would provide more direct evidence of the effects of different hormonal contraceptive methods, in particular DMPA, on the risk of HIV acquisition. In the absence of definitive data, however, women with high HIV risk need access to additional safe and effective contraceptive options, and they need to be counseled about the relative risks and benefits of the available family planning methods.

## Supporting Information

S1 Checklist PRISMA checklist. (DOCX)

S2 Checklist Additional checklist of items specific to individual participant data meta-analyses. (DOCX)

S1 Table Additional information about studies included in the hormonal contraception—HIV individual participant data meta-analysis. (DOCX)

S2 Table Sensitivity analyses from the hormonal contraception—HIV individual participant data meta-analysis, including analyses with studies not included in the primary meta-analysis. (DOCX)

S3 Table Data availability of component datasets for the hormonal contraception—HIV individual participant data meta-analysis. (DOCX)

S1 Text Study protocol. (DOCX)

S2 Text Statistical analysis plan. (DOCX)

## Abbreviations

aHR: adjusted hazard ratio
COC: combined oral contraceptive
DMPA: depot-medroxyprogesterone acetate
HC: hormonal contraception
HR: hazard ratio
HSV-2: herpes simplex virus type 2
IPD: individual participant data
NET-EN: norethisterone enanthate
RCT: randomized controlled trial
VPRP: Vaginal Practices Research Partnership
