# Supplementary material for: Hormonal Contraception and the Risk of HIV Acquisition: An Individual Participant Data Meta-analysis
Source: PLoS Med. 2015 Jan 22;12(1):e1001778. doi: 10.1371/journal.pmed.1001778 (PMC4303292; doi:10.1371/journal.pmed.1001778)
Supplement: S1 Table — (DOCX) [file pmed.1001778.s003.docx]

| Table S1. Additional Information about studies included in the HC-HIV individual participant data meta-analysis | | | | |
| --- | --- | --- | --- | --- |
| **Study Number. Country** | **Short Study Name** | **Author(s) in this IPD Meta-analysis** | **Publication Describing Main Study** | **Published Analyses of Hormonal Contraception and HIV** |
| 1. Kenya | Mombasa Sex Worker Study | RS McClelland | Martin HL et al. *J Infect Dis* 1998;178:1053-1059 [39] | Baeten JM et al. *AIDS* 2007; 21:1771-7 [32] |
| 2. South Africa | Cape Town Cervical Cancer Project | L Myer | Myer L et al. *Am J Epidemiol* 2006;163:552-60 [41] | Myer L et al. *Int J Epidemiol* 2007;36:166-74 [15] |
| 3. Uganda, Zimbabwe | HC-HIV Study | CS Morrison | Morrison CS et al. *AIDS* 2007;21:85-95 [30] | Morrison CS et al. *AIDS* 2007;21:85-95 [30]  Morrison CS et al. *AIDS* 2010;24:1778-81 [31] |
| 4. Kenya | Kibera HIV Study | R Kaul | Kaul R et al. *JAMA* 2004;291:2555-62 [33] | No independent analysis |
| 5. Tanzania | Microbicides Development Program (MDP) | RJ Hayes, SC Francis | Vallely A, et al. *Sex Transm Dis* 2007;34:638-43 [34] | No independent analysis |
| 6. Tanzania | HSV Intervention Study | D Watson-Jones | Watson–Jones D et al. *N Engl J Med* 2008;358:1560-71 [40] | Watson–Jones et al. *AIDS* 2009;23:415-22 [16] |
| 7. Zimbabwe, South Africa | MIRA Study | A van der Straten | Padian NS et al. *Lancet* 2007;370:251-61 [36] | McCoy et al. *AIDS* 2013;27:1001-9 [20] |
| 8. South Africa | Palesa Study | H Rees | Kleinschmidt I et al. *Contraception* 2007;75:461-7 [57] | Kleinschmidt et al. *Contraception* 2007;75:461-7 [57] |
| 9. South Africa | Tshireletso Study | S Delany-Moretlwe | Delany-Moretlwe S et al. RHRU, University of Witwatersrand, S. Africa.available at: <http://www.wrhi.ac.za/Pages/OurStudies.aspx> [37] | No independent analysis |
| 10. South Africa | MDP KZN Microbicide Feasibility Study | N McGrath | McGrath N et al. available at: <http://www.africacentre.ac.za/Portals/0/Researchers/microbicide_protocol_5.0.pdf> [38] | No independent analysis |
| 11. Malawi, Zimbabwe | HIV NET 016 | J Brown | Kumwenda NI et al. *Sex Transm Dis* 2006;33:646-51 [42] | Kumwenda NI et al. *Int J STD AIDS* 2008;19:339-41 [34] |
| 12. South Africa | Carraguard Microbicide Study | BA Friedland, S Karpoff | Skoler-Karpoff S et al. *Lancet* 2008;372:1977-87 [43] | Morrison CS et al. *AIDS* 2012;26:497-504 [18] |
| 13. Uganda | Uganda MRC Microbicide Feasibility Study | RJ Hayes, SC Francis | Vandepitte J et al. *Sex Transm Dis* 2011;38:316-23 [49] | No independent analysis |
| 14. Tanzania | Tanzania MRC Microbicide Feasibility Study | S Kapiga, RJ Hayes | Kapiga S et al. *PLoS One* 2013;8:e68825 [48] | Kapiga S et al. *PLoS One* 2013;8:e68825 [48] |
| 15. East, Southern Africa | Partners in Prevention Study | JM Baeten, R Heffron | Celum C et al*. N Engl J Med* 2010;362:427-39 [59] | Heffron R et al. *Lancet Infect Dis* 2012;12:19-26 [17] |
| 16. East, Southern Africa | MDP 301 Microbicide Trial | S McCormack, A Crook | McCormack S et al. *Lancet* 2010;376(9749):1329-39 [44] | Crook A et al. *Human Reproduction* 2014;29:1810-7 [19] |
| 17. South Africa | CAPRISA 004 Trial | Q Abdool Karim | Abdool Karim Q et al. *Science* 2010;329(5996):1168-74 [5] | No independent analysis |
| 18. East, South Africa | FEMPrEP Trial | L van Damme | van Damme L et al. *N Engl J Med* 2012;367(5):441-22 [6] | No independent analysis |
