# Supplementary material for: Hormonal Contraception and the Risk of HIV Acquisition: An Individual Participant Data Meta-analysis
Source: PLoS Med. 2015 Jan 22;12(1):e1001778. doi: 10.1371/journal.pmed.1001778 (PMC4303292; doi:10.1371/journal.pmed.1001778)
Supplement: S2 Table — (DOCX) [file pmed.1001778.s004.docx]

**Table S2.** Sensitivity analyses from the HC-HIV individual participant data meta-analysis including analyses with studies not included in the primary meta-analysis

|  | | **Time-varying COC** | | | **Time-varying DMPA** | | | **Time-varying NETEN** | |
| --- | --- | --- | --- | --- | --- | --- | --- | --- | --- |
| **Analyses**^a^ | **aHR ((95% CI)** | | **p-value** | **aHR (95% CI)** | | **p-value** | **aHR (95% CI)** | | **p-value** |
| **Overall Results** |  | |  |  | |  |  | |  |
| **One-stage: Stratified Cox** | 1.00 (0.86 - 1.17) | | 0.98 | 1.49 (1.33 - 1.67) | | <.01 | 1.14 (0.95 - 1.38) | | 0.16 |
| **Two-stage: Fixed Effect** | 1.03 (0.88 - 1.20) | | 0.72 | 1.44 (1.28 - 1.62) | | <.01 | 1.12 (0.90 - 1.38) | | 0.32 |
| **Two-stage: Random Effect** | **1.03 (0.88 - 1.20)** | | **0.72** | **1.5 (1.24 - 1.83)** | | **<.01** | **1.24 (0.84 - 1.82)** | | **0.28** |
| **I^2^ and DF^b^** | 0.00 (0 - 38.61) | | DF=16 | 47.33 (8.70 - 69.62) | | DF=17 | 41.52 (0 - 73.10) | | DF=8 |
| **15-24 years of age** |  | |  |  | |  |  | |  |
| **One-stage: Stratified Cox** | 0.84 (0.67 - 1.05) | | 0.12 | 1.28 (1.08 - 1.52) | | <.01 | 0.96 (0.75 - 1.23) | | 0.76 |
| **Two-stage: Fixed Effect** | 0.91 (0.72 - 1.16) | | 0.45 | 1.24 (1.04 - 1.48) | | 0.02 | 0.96 (0.72 - 1.29) | | 0.80 |
| **Two-stage: Random Effect** | 0.91 (0.72 - 1.16) | | 0.45 | 1.25 (1.00- 1.58) | | 0.05 | 0.96 (0.72- 1.29) | | 0.80 |
| **I^2^ and DF^b^** | 0.00 (0 – 39.42) | | DF=14 | 22.41 (0 – 57.36) | | DF=15 | 0.00 (0 – 67.93) | | DF=5 |
| **25+ years of age** |  | |  |  | |  |  | |  |
| **One-stage: Stratified Cox** | 1.12 (0.91 - 1.37) | | 0.29 | 1.64 (1.40 - 1.92) | | <.01 | 1.30 (0.95 - 1.77) | | 0.10 |
| **Two-stage: Fixed Effect** | 1.14 (0.92 - 1.41) | | 0.22 | 1.59 (1.36 - 1.87) | | <.01 | 1.42 (1.02 - 1.99) | | 0.04 |
| **Two-stage: Random Effect** | 1.17 (0.88- 1.56) | | 0.28 | 1.69 (1.25- 2.28) | | <.01 | 1.38 (0.63- 3.04) | | 0.42 |
| **I^2^ and DF^b^** | 30.17 (0 – 63.96) | | DF=12 | 61.53 (33.66 – 77.69) | | DF=15 | 69.55 (32.96 – 86.17) | | DF=6 |
| **HSV-2 negative** |  | |  |  | |  |  | |  |
| **One-stage: Stratified Cox** | 1.08 (0.78 - 1.50) | | 0.64 | 1.48 (1.12 - 1.95) | | 0.01 | 1.16 (0.75 - 1.80) | | 0.50 |
| **Two-stage: Fixed Effect** | 1.15 (0.81 - 1.64) | | 0.44 | 1.50 (1.12 - 1.99) | | <.01 | 1.14 (0.69 - 1.88) | | 0.61 |
| **Two-stage: Random Effect** | 1.23 (0.69 - 2.21) | | 0.49 | 1.61 (1.09 - 2.36) | | 0.02 | 1.14 (0.69 - 1.88) | | 0.61 |
| **I^2^ and DF^b^** | 54.86 (0 – 83.38) | | DF=4 | 26.34 (0 – 68.03) | | DF=6 | 0.00 (0 – 85.84) | | DF=2 |
| **HSV-2 positive** |  | |  |  | |  |  | |  |
| **One-stage: Stratified Cox** | 1.08 (0.89 - 1.30) | | 0.44 | 1.55 (1.33 - 1.80) | | <.01 | 1.43 (1.02 - 2.01) | | 0.04 |
| **Two-stage: Fixed Effect** | 1.09 (0.90 - 1.33) | | 0.39 | 1.53 (1.32 - 1.79) | | 0.01 | 1.61 (1.09 - 2.37) | | 0.02 |
| **Two-stage: Random Effect** | 1.10 (0.87 - 1.39) | | 0.43 | 1.60 (1.18 - 2.17) | | <.01 | 1.61 (1.09 - 2.37) | | 0.02 |
| **I^2^ and DF^b^** | 16.02 (0 – 54.99) | | DF=12 | 65.53 (37.82 – 80.89) | | DF=12 | 0.00 (0 – 70.56) | | DF=2 |

^a^ Adjusted for region, age, married/living with partner, time-varying >1 sex partner, time-varying condom use.

^b^ DF = number of studies - 1

**Table S2.** Sensitivity analyses from the HC-HIV individual participant data meta-analysis including analyses with studies not included in the primary meta-analysis

|  | | **Time-varying COC** | | | **Time-varying DMPA** | | | | **Time-varying NETEN** | |
| --- | --- | --- | --- | --- | --- | --- | --- | --- | --- | --- |
| **Analyses**^a^ | **HR ((95% CI)** | | **p-value** | **HR (95% CI)** | | **p-value** | | **HR (95% CI)** | | **p-value** |
| **Higher risk of bias** |  | |  |  | |  |  | | |  |
| **One-stage: Stratified Cox** | 1.13 (0.92 - 1.40) | | 0.25 | 1.80 (1.53 - 2.11) | | <.01 | 1.54 (1.21 - 1.96) | | | <.01 |
| **Two-stage: Fixed Effect** | 1.16 (0.93 - 1.45) | | 0.19 | 1.78 (1.51 - 2.11) | | <.01 | 1.50 (1.14 - 1.96) | | | 0.01 |
| **Two-stage: Random Effect** | 1.16 (0.93- 1.45) | | 0.19 | 1.73 (1.39- 2.16) | | <.01 | 1.50 (1.14- 1.96) | | | <.01 |
| **I^2^ and DF^b^** | 0.00 (0 – 32.25) | | DF=11 | 19.16 (0 – 57.39) | | DF=12 | 0.00 (N/A) | | | DF=6 |
| **Lower risk of bias** |  | |  |  | |  |  | | |  |
| **One-stage: Stratified Cox** | 0.90 (0.72 - 1.11) | | 0.33 | 1.22 (1.03 - 1.43) | | 0.02 | 0.79 (0.58 - 1.09) | | | 0.15 |
| **Two-stage: Fixed Effect** | 0.91 (0.73 - 1.14) | | 0.40 | 1.18 (1.00 - 1.39) | | 0.05 | 0.67 (0.47 - 0.96) | | | 0.03 |
| **Two-stage: Random Effect** | 0.91 (0.73- 1.14) | | 0.40 | 1.22 (0.99- 1.50) | | 0.06 | 0.67 (0.47- 0.96) | | | 0.03 |
| **I^2^ and DF^b^** | 0.00 (0 – 77.53) | | DF=4 | 28.50 (0 – 72.15) | | DF=4 | 0.00 (N/A) | | | DF=1 |
| **Time-varying Pregnancy** |  | |  |  | |  |  | | |  |
| **One-stage: Stratified Cox** | 1.00 (0.85 - 1.17) | | 0.95 | 1.50 (1.33 - 1.70) | | <.01 | 1.14 (0.94 - 1.39) | | | 0.18 |
| **Two-stage: Fixed Effect** | 1.01 (0.85 - 1.19) | | 0.94 | 1.43 (1.26 - 1.62) | | <.01 | 1.14 (0.91 - 1.43) | | | 0.24 |
| **Two-stage: Random Effect** | 1.01 (0.85- 1.19) | | 0.94 | 1.48 (1.18- 1.85) | | <.01 | 1.32 (0.79- 2.19) | | | 0.28 |
| **I^2^ and DF^b^** | 0.00 (0 – 52.95) | | DF=13 | 52.24 (13.84 – 73.53) | | DF=14 | 59.58 (16.85 – 82.46) | | | DF=6 |
| **Censoring pregnant visits** |  | |  |  | |  | |  | |  |
| **One-stage: Stratified Cox** | 0.97 (0.82 - 1.14) | | 0.71 | 1.53 (1.35 - 1.73) | | <.01 | | 1.16 (0.95 - 1.41) | | 0.15 |
| **Two-stage: Fixed Effect** | 0.99 (0.83 - 1.17) | | 0.87 | 1.48 (1.30 - 1.68) | | <.01 | | 1.16 (0.92 - 1.45) | | 0.21 |
| **Two-stage: Random Effect** | 0.99 (0.83- 1.17) | | 0.87 | 1.58 (1.25- 2.01) | | <.01 | | 1.29 (0.84- 1.99) | | 0.25 |
| **I^2^ and DF^b^** | 0.00 (0 – 55.98) | | DF=12 | 58.00 (26.77 – 75.91) | | DF=15 | | 49.20 (0 – 77.39) | | DF=7 |
| **Excluding women ever pregnant** |  | |  |  | |  | |  | |  |
| **One-stage: Stratified Cox** | 1.00 (0.85 - 1.18) | | 0.99 | 1.44 (1.27 - 1.63) | | <.01 | | 1.10 (0.91 - 1.34) | | 0.33 |
| **Two-stage: Fixed Effect** | 1.00 (0.84 - 1.19) | | 0.98 | 1.40 (1.23 - 1.59) | | <.01 | | 1.12 (0.90 - 1.41) | | 0.31 |
| **Two-stage: Random Effect** | 1.00 (0.84 -1.19) | | 0.98 | 1.51 (1.18- 1.93) | | <.01 | | 1.25 (0.83- 1.89) | | 0.29 |
| **I^2^ and DF^b^** | 0.00 (0 – 53.76) | | DF=12 | 60.67 (32.00 – 77.26) | | DF=15 | | 44.61 (0 – 75.52) | | DF=7 |

^a^ Adjusted for region, age, married/living with partner, time-varying >1 sex partner, time-varying condom use.

^b^ DF = number of studies - 1

**Table S2.** Sensitivity analyses from the HC-HIV individual participant data meta-analysis including analyses with studies not included in the primary meta-analysis

|  | | **Time-varying COC** | | **Time-varying DMPA** | | | **Time-varying NETEN** | |
| --- | --- | --- | --- | --- | --- | --- | --- | --- |
| **Analyses**^a^ | **HR ((95% CI)** | | **p-value** | **HR (95% CI)** | **p-value** | **HR (95% CI)** | | **p-value** |
| **Censoring at HC switch (no HC switch)** | | | | | | | | |
| **One-stage: Stratified Cox** | 0.98 (0.80 - 1.20) | | 0.85 | 1.52 (1.31 - 1.75) | <.01 | 1.23 (0.97 - 1.56) | | 0.08 |
| **Two-stage: Fixed Effect** | 1.01 (0.81 - 1.25) | | 0.93 | 1.48 (1.28 - 1.72) | <.01 | 1.18 (0.90 - 1.55) | | 0.22 |
| **Two-stage: Random Effect** | 1.01 (0.81- 1.25) | | 0.93 | 1.51 (1.20- 1.89) | <.01 | 1.28 (0.82- 1.99) | | 0.27 |
| **I^2^ and DF^b^** | 0.00 (0 - 29.48) | | DF=12 | 41.90 (0 – 68.47) | DF=14 | 44.28 (0 – 78.00) | | DF=5 |
| **Studies with Specified Injectables** |  | |  |  |  |  | |  |
| **One-stage: Stratified Cox** | 1.06 (0.90 - 1.26) | | 0.48 | 1.62 (1.42 - 1.85) | <.01 | 1.22 (1.00 - 1.49) | | 0.05 |
| **Two-stage: Fixed Effect** | 1.07 (0.90 - 1.28) | | 0.46 | 1.55 (1.36 - 1.78) | <.01 | 1.11 (0.90 - 1.38) | | 0.33 |
| **Two-stage: Random Effect** | 1.07 (0.90 - 1.28) | | 0.46 | 1.61 (1.28 - 2.03) | <.01 | 1.28 (0.82 - 2.00) | | 0.28 |
| **I^2^ and DF^2^** | 0.00 (0 – 50.96) | | DF=12 | 48.79 (4.92 – 72.41) | DF=13 | 56.11 (0 – 81.17) | | DF=6 |
| **Censoring at time of condom use** |  | |  |  |  |  | |  |
| **One-stage: Stratified Cox** | 0.95 (0.65 - 1.39) | | 0.81 | 1.45 (1.10 - 1.92) | 0.01 | 1.05 (0.70 - 1.58) | | 0.81 |
| **Two-stage: Fixed Effect** | 1.08 (0.71 - 1.65) | | 0.72 | 1.44 (1.09 - 1.92) | 0.01 | 1.00 (0.66 - 1.51) | | 0.98 |
| **Two-stage: Random Effect** | 1.08 (0.71- 1.65) | | 0.72 | 1.6 (1.11- 2.31) | 0.01 | 1.32 (0.65- 2.69) | | 0.45 |
| **I^2^ and DF^b^** | 0.00 (0 – 62.45) | | DF=5 | 23.35 (0 – 62.80) | DF=9 | 46.93 (0 – 79.03) | | DF=5 |
| **Eastern Africa** |  | |  |  |  |  | |  |
| **One-stage: Stratified Cox** | 1.55 (1.19 - 2.02) | | <.01 | 2.13 (1.72 - 2.63) | <.01 | N/A | |  |
| **Two-stage: Fixed Effect** | 1.58 (1.19 - 2.09) | | <.01 | 2.09 (1.68 - 2.59) | <.01 | N/A | |  |
| **Two-stage: Random Effect** | 1.58 (1.19 - 2.09) | | <.01 | 2.09 (1.68 - 2.60) | <.01 | N/A | |  |
| **I^2^ and DF^b^** | 0.00 (0 – 29.28) | | DF=8 | 0.51 (0 – 7.41) | DF=8 | N/A | |  |
| **Southern Africa** |  | |  |  |  |  | |  |
| **One-stage: Stratified Cox** | 0.79 (0.60 - 1.05) | | 0.10 | 0.94 (0.71 - 1.25) | 0.66 | 1.13 (0.34 - 3.73) | | 0.84 |
| **Two-stage: Fixed Effect** | 0.81 (0.61 - 1.06) | | 0.13 | 0.94 (0.70 - 1.26) | 0.68 | 1.18 (0.33 - 4.25) | | 0.80 |
| **Two-stage: Random Effect** | 0.81 (0.61 - 1.06) | | 0.13 | 0.94 (0.70 - 1.26) | 0.68 | 1.18 (0.33 - 4.25) | | 0.80 |
| **I^2^ and DF^b^** | 0.00 (N/A) | | DF=4 | 0.00 (0 – 39.90) | DF=4 | 0.00 (N/A) | | DF=0 |

^a^ Adjusted for region, age, married/living with partner, time-varying >1 sex partner, time-varying condom use.

^b^ DF = number of studies - 1

**Table S2.** Sensitivity analyses from the HC-HIV individual participant data meta-analysis including analyses with studies not included in the primary meta-analysis

|  | | **Time-varying COC** | | **Time-varying DMPA** | | | **Time-varying NETEN** | |
| --- | --- | --- | --- | --- | --- | --- | --- | --- |
| **Analyses**^a^ | **HR ((95% CI)** | | **p-value** | **HR (95% CI)** | **p-value** | **HR (95% CI)** | | **p-value** |
| **South Africa** |  | |  |  |  |  | |  |
| **One-stage: Stratified Cox** | 0.82 (0.64 - 1.05) | | 0.12 | 1.36 (1.16 - 1.59) | <.01 | 1.07 (0.88 - 1.32) | | 0.50 |
| **Two-stage: Fixed Effect** | 0.83 (0.63 - 1.08) | | 0.16 | 1.30 (1.11 - 1.53) | <.01 | 1.06 (0.85 - 1.32) | | 0.63 |
| **Two-stage: Random Effect** | 0.83 (0.63 - 1.08) | | 0.16 | 1.3 (1.11 - 1.53) | <.01 | 1.17 (0.81 - 1.70) | | 0.40 |
| **I^2^ and DF^b^** | 0.00 (N/A) | | DF=7 | 0.00 (0 – 59.79) | DF=9 | 38.33 (0 – 72.83) | | DF=7 |
| **Low Incidence population** |  | |  |  |  |  | |  |
| **One-stage: Stratified Cox** | 1.04 (0.86 - 1.26) | | 0.71 | 1.59 (1.37 - 1.85) | <.01 | 1.16 (0.94 - 1.44) | | 0.17 |
| **Two-stage: Fixed Effect** | 1.01 (0.83 - 1.23) | | 0.92 | 1.50 (1.29 - 1.75) | <.01 | 1.07 (0.85 - 1.34) | | 0.57 |
| **Two-stage: Random Effect** | 1.01 (0.82 - 1.25) | | 0.92 | 1.64 (1.23 - 2.19) | <.01 | 1.15 (0.66 - 1.99) | | 0.63 |
| **I^2^ and DF^b^** | 3.50 (0 – 31.62) | | DF=7 | 60.95 (15.17 – 82.02) | DF=7 | 74.33 (28.23 – 90.82) | | DF=3 |
| **High Incidence population** |  | |  |  |  |  | |  |
| **One-stage: Stratified Cox** | 1.03 (0.79 - 1.33) | | 0.85 | 1.32 (1.10 - 1.59) | <.01 | 1.88 (0.92 - 3.82) | | 0.08 |
| **Two-stage: Fixed Effect** | 1.06 (0.81 - 1.37) | | 0.68 | 1.35 (1.12 - 1.62) | <.01 | 1.59 (0.75 - 3.38) | | 0.23 |
| **Two-stage: Random Effect** | 1.06 (0.81 - 1.37) | | 0.68 | 1.34 (0.98 - 1.83) | 0.06 | 1.59 (0.75 - 3.38) | | 0.23 |
| **I^2^ and DF^b^** | 0.00 (0 – 61.85) | | DF=6 | 45.86 (0 – 76.03) | DF=7 | 0.00 (N/A) | | DF=2 |
| **Transactional sex** |  | |  |  |  |  | |  |
| **One-stage: Stratified Cox** | 1.34 (0.98 - 1.84) | | 0.07 | 1.82 (1.41 - 2.35) | <.01 | 0.73 (0.09 - 6.19) | | 0.77 |
| **Two-stage: Fixed Effect** | 1.51 (1.09 - 2.10) | | 0.01 | 1.82 (1.41 - 2.36) | <.01 | 0.92 (0.06 - 13.28) | | 0.95 |
| **Two-stage: Random Effect** | 1.51 (1.09 - 2.10) | | 0.01 | 1.76 (1.29 - 2.40) | <.01 | 0.92 (0.06 - 13.43) | | 0.95 |
| **I^2^ and DF^b^** | 0.00 (0 – 59.10) | | DF=6 | 12.53 (0 – 54.39) | DF=8 | N/A | | DF=0 |
| **No Transactional sex** |  | |  |  |  |  | |  |
| **One-stage: Stratified Cox** | 0.92 (0.78 - 1.09) | | 0.33 | 1.41 (1.24 - 1.61) | <.01 | 1.10 (0.91 - 1.33) | | 0.34 |
| **Two-stage: Fixed Effect** | 0.96 (0.80 - 1.15) | | 0.68 | 1.37 (1.20 - 1.57) | <.01 | 1.12 (0.90 - 1.39) | | 0.30 |
| **Two-stage: Random Effect** | 0.96 (0.80 - 1.15) | | 0.68 | 1.45 (1.16 - 1.80) | <.01 | 1.25 (0.84 - 1.85) | | 0.27 |
| **I^2^ and DF^b^** | 0.00 (0 – 32.90) | | DF=11 | 43.79 (0 – 69.40) | DF=14 | 41.86 (0 – 73.24) | | DF=8 |

^a^ Adjusted for region, age, married/living with partner, time-varying >1 sex partner, time-varying condom use.

^b^ DF = number of studies - 1

**Table S2.** Sensitivity analyses from the HC-HIV individual participant data meta-analysis including analyses with studies not included in the primary meta-analysis

|  | | **Time-varying COC** | | | **Time-varying DMPA** | | | | **Time-varying NETEN** | |
| --- | --- | --- | --- | --- | --- | --- | --- | --- | --- | --- |
| **Analyses**^a^ | **HR ((95% CI)** | | **p-value** | | **HR (95% CI)** | | **p-value** | **HR (95% CI)** | | **p-value** |
| **RCT studies only** |  | |  | |  | |  |  | |  |
| **One-stage: Stratified Cox** | 0.93 (0.77 - 1.13) | | 0.47 | | 1.43 (1.24 - 1.64) | | <.01 | 1.07 (0.87 - 1.31) | | 0.52 |
| **Two-stage: Fixed Effect** | 0.97 (0.79 - 1.18) | | 0.75 | 1.38 (1.19 - 1.59) | | <.01 | | 1.08 (0.86 - 1.35) | | 0.51 |
| **Two-stage: Random Effect** | 0.97 (0.77 - 1.22) | | 0.81 | 1.43 (1.13 - 1.81) | | <.01 | | 1.22 (0.71 - 2.11) | | 0.47 |
| **I^2^ and DF^b^** | 8.30 (0 – 47.62) | | DF=8 | 44.76 (0 – 74.50) | | DF=8 | | 60.78 (3.78 – 84.01) | | DF=5 |
| **Cohort studies only** |  | |  |  | |  | |  | |  |
| **One-stage: Stratified Cox**  **Two-stage: Fixed Effect** | 1.17 (0.92 - 1.50)  1.13 (0.88 - 1.45) | | 0.20  0.33 | 1.54 (1.26 - 1.89)  1.59 (1.30 - 1.95) | | <.01  <.01 | | 1.89 (1.02 - 3.49)  1.47 (0.76 - 2.84) | | 0.04  0.25 |
| **Two-stage: Random Effect** | 1.13 (0.88 - 1.45) | | 0.33 | 1.58 (1.11 - 2.24) | | 0.01 | | 1.47 (0.76 - 2.84) | | 0.25 |
| **I^2^ and DF^b^** | 0.00 (0 – 26.83) | | DF=7 | 51.45 (0 - 77.30) | | DF=8 | | 0.00 (0 – 14.10) | | DF=2 |
| **Specific covariates for each study (‘chosen’ model)** | | | | | | | | | | |
| **One-stage: Stratified Cox**  **Two-stage: Fixed Effect** | N/A  1.07 (0.91 - 1.25) | | 0.43 | N/A  1.46 (1.29 - 1.64) | | <.01 | | N/A  1.26 (1.01 - 1.57) | | 0.04 |
| **Two-stage: Random Effect** | 1.07 (0.91 - 1.25) | | 0.43 | 1.52 (1.27 - 1.82) | | <.01 | | 1.27 (0.99 - 1.61) | | 0.06 |
| **I^2^ and DF^b^** | 0.00 (0 – 26.80) | | DF=16 | 38.86 (0 – 65.24) | | DF=17 | | 5.14 (0 - 38.84) | | DF=7 |
| **Adding new study (Chirenje (61))** |  | |  |  | |  | |  | |  |
| **One-stage: Stratified Cox** | N/A | |  | N/A | |  | | N/A | |  |
| **Two-stage: Fixed Effect** | 1.00 (0.86 - 1.16) | | 0.99 | 1.44 (1.28 - 1.61) | | <.01 | | 1.12 (0.90 - 1.38) | | 0.32 |
| **Two-stage: Random Effect** | 1.00 (0.86 - 1.16) | | 0.99 | 1.48 (1.24 - 1.77) | | <.01 | | 1.24 (0.84 - 1.82) | | 0.28 |
| **I^2^ and DF^b^** | 0.00 (0 – 43.14) | | DF=17 | 44.41 (0 – 67.65) | | DF=18 | | 41.50 (0 – 73.09) | | DF=8 |

^a^ Adjusted for region, age, married/living with partner, time-varying >1 sex partner, time-varying condom use.

^b^ DF = number of studies – 1
