# Supplementary material for: Hormonal Contraception and the Risk of HIV Acquisition: An Individual Participant Data Meta-analysis
Source: PLoS Med. 2015 Jan 22;12(1):e1001778. doi: 10.1371/journal.pmed.1001778 (PMC4303292; doi:10.1371/journal.pmed.1001778)
Supplement: S3 Table — (DOCX) [file pmed.1001778.s005.docx]

**Table S3. Data availability of component datasets for the HC-HIV individual participant data meta-analysis**

**Data Available upon Request**

| Name of Study | Study Website | Contact Person | Email |
| --- | --- | --- | --- |
| 1. Mombasa Sex Worker Study | N/A | Scott McClelland | [mcclell@uw.edu](file:///C:\Users\cmorrison\AppData\Local\Microsoft\Windows\Temporary%20Internet%20Files\Content.Outlook\NPHYNQGN\mcclell@uw.edu) |
| 2. Cape Town Cervical Cancer Project | N/A | Landon Myer | [landon.myer@uct.ac.za](mailto:landon.myer@uct.ac.za) |
| 3. HC-HIV Study | N/A | Charles Morrison | [cmorrison@fhi360.org](mailto:cmorrison@fhi360.org) |
| 4. Kibera HIV Study | N/A | Rupert Kaul | [rupert.kaul@utoronto.ca](mailto:rupert.kaul@utoronto.ca) |
| 5. Microbicides Development Program (Mwanza Feasibility)* | N/A | Saidi Kapiga | [Saidi.Kapiga@lshtm.ac.uk](mailto:Saidi.Kapiga@lshtm.ac.uk) |
| 6. HSV Intervention Study* | N/A | Saidi Kapiga | [Saidi.Kapiga@lshtm.ac.uk](mailto:Saidi.Kapiga@lshtm.ac.uk) |
| 7. MIRA Study | N/A | Ariane van der Straten | [ariane@rti.org](mailto:ariane@rti.org) |
| 8. Palesa Study | N/A | Helen Rees  Sinead Delany-Moretlwe | [hrees@wrhi.ac.za](file:///C:\Users\cmorrison\AppData\Local\Microsoft\Windows\Temporary%20Internet%20Files\Content.Outlook\NPHYNQGN\hrees@wrhi.ac.za)  [sdelany@wrhi.ac.za](file:///C:\Users\cmorrison\AppData\Local\Microsoft\Windows\Temporary%20Internet%20Files\Content.Outlook\NPHYNQGN\sdelany@wrhi.ac.za) |
| 9. Tshireletso Study | N/A | Helen Rees  Sinead Delany-Moretlwe | [hrees@wrhi.ac.za](file:///C:\Users\cmorrison\AppData\Local\Microsoft\Windows\Temporary%20Internet%20Files\Content.Outlook\NPHYNQGN\hrees@wrhi.ac.za)  [sdelany@wrhi.ac.za](file:///C:\Users\cmorrison\AppData\Local\Microsoft\Windows\Temporary%20Internet%20Files\Content.Outlook\NPHYNQGN\sdelany@wrhi.ac.za) |
| 10. MDP KZN Microbicide Feasibility Study | N/A | Nuala McGrath | [N.McGrath@soton.ac.uk](file:///C:\Users\cmorrison\AppData\Local\Microsoft\Windows\Temporary%20Internet%20Files\Content.Outlook\NPHYNQGN\N.McGrath@soton.ac.uk) |
| 11. HIV NET 016 | N/A | Lynda Emel | [lemel@scharp.org](mailto:lemel@scharp.org) |
| 12. Carraguard Microbicide Trial | N/A | Barbara Friedland  Naomi Rutenberg | [bfriedland@popcouncil.org](file:///C:\Users\cmorrison\AppData\Local\Microsoft\Windows\Temporary%20Internet%20Files\Content.Outlook\NPHYNQGN\bfriedland@popcouncil.org)  [nrutenberg@popcouncil.org](mailto:nrutenberg@popcouncil.org) |
| 13. Uganda MRC Microbicide Feasibility Study* | N/A | Anatoli Kamali | [Anatoli.Kamali@mrcuganda.org](file:///C:\Users\cmorrison\AppData\Local\Microsoft\Windows\Temporary%20Internet%20Files\Content.Outlook\NPHYNQGN\Anatoli.Kamali@mrcuganda.org) |
| 14. Tanzania MRC Microbicide Feasibility Study* | N/A | Saidi Kapiga | [Saidi.Kapiga@lshtm.ac.uk](mailto: Saidi.Kapiga@lshtm.ac.uk) |
| 15. Partners in Prevention Trial | [http://depts.washington.edu/uwicrc/research/studies/pip_transmission](http://depts.washington.edu/uwicrc/research/studies/pip_transmission.html) | Jared Baeten | [jbaeten@uw.edu](file:///C:\Users\cmorrison\AppData\Local\Microsoft\Windows\Temporary%20Internet%20Files\Content.Outlook\NPHYNQGN\jbaeten@uw.edu) |
| 16. MDP 301 Microbicide Trial | <http://www.mdp.mrc.ac.uk/> | Angela Crook | [mrcctu.ctuenquiries@ucl.ac.uk](mailto:mrcctu.ctuenquiries@ucl.ac.uk) |
| 17. CAPRISA 004 Trial | [http://www.caprisa.org](http://www.caprisa.org/) | Anneke Grobler | [Anneke.grobler@caprisa.org](mailto:Anneke.grobler@caprisa.org) |
| 18. FEMPrEP Trial | <http://femprep.fhi360.org/> | Jennifer Deese | [jdeese@fhi360.org](file:///C:\Users\tcolter\AppData\Local\Microsoft\Windows\Temporary%20Internet%20Files\Content.Outlook\56Z0X303\jdeese@fhi360.org) |

*The Tanzanian (MITU) and the Ugandan (MRC Uganda) data may be made available on request as per the respective organizations Data Sharing Policy guidelines; interested parties must submit a Data Access Application form to the Data Access Committee. At MITU, requests should be made to Saidi Kapiga (Chair of the committee). For the MRC Uganda requests should be made to Anatoli Kamali (Programme Head for this project).
