# Supplementary material for: Hormonal Contraception and the Risk of HIV Acquisition: An Individual Participant Data Meta-analysis
Source: PLoS Med. 2015 Jan 22;12(1):e1001778. doi: 10.1371/journal.pmed.1001778 (PMC4303292; doi:10.1371/journal.pmed.1001778)
Supplement: S1 Text — (DOCX) [file pmed.1001778.s006.docx]

**Hormonal Contraception and the Risk of HIV Acquisition: An Individual Participant Data Meta-Analysis**

**Study # 10263**

**Funded by:** National Institutes of Health, the Eunice Kennedy Shriver National Institute of Child Health & Human Development award number R21HD069192

**FHI 360 Principal Investigator:** Charles Morrison, PhD

**FHI 360 Co-Investigator: Senior Biostatistician:** Pai Lien Chen, PhD

**University of Bern Consultant:** Nicola Low, MD, PhD

**FHI 360 Associate Biostatistician:** Cynthia Kwok, MS

**Collaborators:** J. Baeten, S. McClelland, R. Heffron, L. Myer, R. Kaul, S. Francis, D. Watson-Jones, R. Hayes, A. van der Straten, H Rees, S. Delany-Moretlwe, N. McGrath, J. Brown, J. van de Wijgert, B. Friedland, S. Skoler-Karpoff, S. Kapiga, S. McCormack, A. Crook, L. Van Damme, A. Grobler, Q. Karim

**Hormonal Contraception and the Risk of HIV Acquisition: An Individual Participant Data Meta-Analysis**

**TABLE OF CONTENTS**

**STUDY TEAM**  1

**STUDY SUMMARY** 3

**LIST OF ABBREVIATIONS AND ACRONYMS** 4

**1 INTRODUCTION** 5

**2 OBJECTIVES AND HYPOTHESES** 5

**3 METHODS**…… 6

3.1 Overview of Studies … 6

3.2 Inclusion and Exclusion criteria for meta-analysis … 6

3.2 Types of Participants … 8

3.4 Exposures and Outcomes … 8

3.5 Censoring … 8

3.6 Assessment of the risk of bias in individual studies … 8

1. **DATA COLLECTION AND MANAGEMENT** 9

**5 ANALYSIS** 10

- 1. Descriptive Analysis 10
  2. Assessment of Confounding 10
  3. Effect Modification 11

5.4 Pregnancy 12

5.5 Meta-analysis 12

5.6 Study Limitations and Sensitivity Analyses 13

5.6 Supplementary Analyses 14

**6 REFERENCES** 15

**APPENDICES**

Table 1. VPRP Studies 17

Table 2. Additional studies to be added to VPRP for meta-analysis 18

Table 3. Planned analyses and analysis populations 19

**Hormonal Contraception and the Risk of HIV Acquisition: An Individual Participant Data Meta-Analysis**

**Study # 10263**

**STUDY SUMMARY**

**Purpose:** To conduct an individual participant data (IPD) meta-analysis of prospective studies to determine whether hormonal contraception use increases women’s risk of acquiring HIV infection.

**Design:** Meta-analysis of individual participant data from 10 VPRP studies plus 8 additional prospective studies using both one- and two-stage meta-analysis approaches.

**Study Population:** An estimated 38,000 women from the general population and groups of women at high-risk of HIV infection from selected prospective studies in sub-Saharan Africa.

**Primary Objectives:** 1.) To evaluate the effect of hormonal contraceptive use including depo-medroxyprogesterone acetate (DMPA), combined oral contraceptives (OCs), and norethisterone enanthate (Net-En) on the risk of HIV acquisition among young women (ages 15-24 years).

2.) To evaluate the effect of hormonal contraceptive use (DMPA, OCs, Net-En) on the risk of HIV acquisition among older women (ages 25-49 years).

3.) To evaluate the effect of hormonal contraceptive use (DMPA, OCs, Net-En) on the risk HIV acquisition across both age groups.

4.) To evaluate whether HSV-2 infection status alters the effect of hormonal contraception (DMPA, OC, and Net-En) on the risk of HIV acquisition.

**Secondary Objectives:** 1.) To directly compare the risks of HIV acquisition among women using the three study contraceptive methods (DMPA, OCs, Net-En).

2.) To examine whether measured behavioral covariates explain any potentially increased risk of HIV acquisition among young women using DMPA, OCs, and Net-En.

**LIST OF ABBREVIATIONS AND ACRONYMS**

CDC Centers for Disease Control and Prevention

COC combined oral contraceptive

CT *Chlamydia trachomatis*

DMPA Depo-medroxyprogesterone acetate or Depo-Provera

EDCTP European and Developing Countries Clinical Trials Partnership

ELISA enzyme-linked immunosorbent assay

GC gonorrhea

HC hormonal contraception

HC-HIV Study Hormonal Contraception and Risk of HIV Acquisition Study

HIV human immunodeficiency virus

HSV-2 herpes simplex virus type 2

IM intramuscular

IPD individual-participant data

ISPM The Institute of Social and Preventative Medicine at the University of Bern

IUD intrauterine device

Net-En Norethisterone enanthate

OC oral contraceptive

PCR polymerase chain reaction

PID pelvic inflammatory disease

POP progestin-only pill

STI sexually transmitted infection

STROBE strengthening the reporting of observational studies in epidemiology

VPRP Vaginal Practices Research Partnership

WRHI Wits Reproductive Health and HIV Institute

**1. BACKGROUND**

The FHI 360 Clinical Sciences and Biostatistics Departments are conducting an individual participant data (IPD) meta-analysis of the association between use of hormonal contraception and HIV acquisition. The Institute of Social and Preventive Medicine (ISPM) at the University of Bern, Switzerland, is collaborating with FHI 360 on this meta-analysis. The ISPM is the coordinating center for the Vaginal Practices Research Partnership (VPRP) which is a partnership among numerous research groups with longitudinal datasets to investigate a possible association between various vaginal practices and HIV acquisition. The complete set of studies that participated in the VPRP is listed in Table 1. The main report from the VPRP has been published previously (1).

Evidence on whether hormonal contraception (HC) use alters a woman’s risk of HIV acquisition is mixed. For ethical and logistical reasons (i.e. cost and difficulty of randomizing women to various contraceptive methods), no randomized trials of HC use and HIV acquisition have been completed. The associations between different HC methods and HIV have therefore been examined in prospective cohort studies. To our knowledge, analyses have been conducted on sixteen prospective datasets to examine the relationship between depo-medroxyprogesterone acetate (DMPA) and HIV acquisition (2-17). Six found a significantly increased HIV risk associated with DMPA (2,3, 5, 8, 11, 13). Three analyses of longitudinal data have found no significant relationship between norethisterone enanthate (Net-En) and HIV (7, 12, 14). No meta-analyses have been conducted of the relationship between injectable contraceptives (DMPA or Net-En) and HIV acquisition.

The Hormonal Contraception and Risk of HIV Acquisition (HC-HIV) study, led by FHI 360, has been the largest study specifically designed to examine hormonal contraception and HIV acquisition and was one of the VPRP component datasets. In the HC-HIV study, we found that the association between HC and HIV was modified by age and by HSV-2 status (11, 18). Thus, we are interested in exploring the effect of age and HSV-2 on the HC-HIV relationship in this IPD meta-analysis.

Because the studies participating in the VPRP were all judged to be of high quality, all have prospective data on hormonal contraceptive use and HIV acquisition, and because many of the variables have been harmonized between the VPRP studies, we decided to conduct an individual participant data meta-analysis using the component VPRP datasets.

**2. OBJECTIVES AND HYPOTHESES**

This study will be a meta-analysis of individual participant data (IPD) from prospective longitudinal studies. The main aim of the IPD meta-analysis is to determine whether hormonal contraception, including combined oral contraceptives (OCs) and the progestin-only injectables DMPA and Net-En increase women’s risk of acquiring HIV infection. Using data from 18 longitudinal studies (ten from the VPRP and eight additional studies), we will compare the rate of HIV acquisition among women who use OCs, DMPA and Net-En with the rate among women who do not use hormonal contraception.

More specifically, we will investigate the following objectives:

1. To determine whether use of different hormonal contraceptives (COCs, DMPA and Net-En, separately) increases the risk of HIV acquisition compared to women not using hormonal contraception.
   1. To determine whether use of different hormonal contraceptives (COCs, DMPA and Net-En, separately) increases the risk of HIV acquisition among young women (ages 15-24 years).
   2. To determine whether use of different hormonal contraceptives (COCs, DMPA and Net-En, separately) increases the risk of HIV acquisition among older women (ages 25-49 years).
   3. To evaluate whether HSV-2 infection status (across both age groups) modifies the effect of hormonal contraception (DMPA, OC, and Net-En, separately) on the risk of HIV acquisition and to determine whether different hormonal contraceptives increase the risk of HIV acquisition among HSV-2 negative and HSV-2 positive women.
2. To compare the risks of HIV acquisition among the three hormonal contraceptive groups (DMPA, OC and Net-En users).

**3. METHODS**

**3.1. Overview of Studies**

We included randomized controlled trials of non-contraceptive HIV prevention interventions and cohort studies that contain prospectively-collected data on both hormonal contraceptive use and incident HIV-1 infections. Only studies from Sub-Saharan Africa (where most studies have been conducted) were included.

**3.2. Inclusion and exclusion criteria for studies to participate in the meta-analysis**

The inclusion and exclusion criteria for studies to participate in the IPD meta-analysis are as follows:

**Inclusion Criteria**

- Measured HIV prospectively at multiple time points with a testing interval of 6 months or less
- Measured HIV using a standardized testing algorithm;
- Measured hormonal contraceptive use prospectively at multiple time points with a measurement interval of 6 month or less
- Measured hormonal contraceptive use using a standardized questionnaire
- Included women between the ages of 15-49 years
- Included women who used injectable contraception
- Included at least 15 incident HIV infections in the dataset
- Measured important covariates including (at a minimum) age, condom use, and number of sexual partners.

**Exclusion criteria**

The following types of studies will be excluded:

- Where either HIV infection or hormonal contraceptive use were not measured prospectively and at multiple time points or with a testing interval of more than 6 months;
- Hormonal contraceptive use is not measured prospectively using standardized data collection forms or has a measurement interval of more than 6 months;
- Where there is a significant amount (>5%) of missing HIV infection or hormonal contraceptive use data;
- Studies with small numbers of women (or person-time) using hormonal contraception;
- Studies where the frequency of scheduled follow-up visits is longer than 6 months apart;
- For studies that had an intervention arm where anti-retroviral drugs were provided (for prevention of HIV infection), women assigned to the intervention arm were excluded

The selection of studies into the meta-analysis was essentially opportunistic. This is partially due to the fact that most studies that have a published estimate of the effect of HC on HIV were not designed to measure the association and many had important weaknesses in relation to measuring the HC and HIV relationship. Conversely, a number of other studies that have not published estimates of the effect of HC on HIV have data that are better suited for estimating this association. We included all ten studies included in the VPRP IPD meta-analysis (Table 1) in the HC-HIV meta-analysis: they met all eligibility criteria including measuring hormonal contraception and HIV infection prospectively and were judged to be of good methodological quality, based on published reports (1). We also identified eight additional eligible studies - the Carraguard Microbicide Study, the Partners in Prevention HSV/HIV Transmission Study (and the companion Couple Observational Study), the CDC-WRHR Palesa study, the EDCTP Microbicide Feasibility Studies in Uganda and Tanzania and the MDP 301 study (Table 2), the FEM-PrEP oral PrEP trial, the CAPRISA 004 Tenofovir gel trial, and the MDP 301 Microbicide trial. While these additional studies were largely a convenience sample, each of the studies meets the inclusion criteria and has some important characteristics. For example the Carraguard study is very large (270 incident HIV infections), included large numbers of DMPA, OC and Net-En users and also included many women 16-24 years of age – an important age subgroup for this meta-analysis. The Palesa study, despite its relatively small size, was one of only three studies specifically designed to examine the HC-HIV association and includes both DMPA and Net-En users. The Partners in Prevention HSV/HIV Transmission Study with its observational companion study are the only serodiscordant couples study included and thus the only study where women were known to be exposed to an HIV-infected partner (thus eliminating an important source of potential confounding). The MDP 301 trial is the largest HIV prevention trial that measured hormonal contraception prospectively. The other two studies also meet the study inclusion criteria and were conducted from investigators already participating in the VPRP.

Of the ten original studies in the VPRP dataset four have published estimates of the relationship between hormonal contraception (either injectables or COCs) and HIV acquisition (8, 9, 11, 14, 18). Of the eight additional studies included in this meta-analysis, three have published estimates of the relationship between hormonal contraception and HIV acquisition (5, 7, 12).

**3.1.2. Types of participants**

Study populations where heterosexual transmission is thought to be the predominant mode of HIV transmission will be included. Studies include both women from the general population and groups of women identified at high-risk of HIV infection such as sex workers and HIV-negative women who are part of HIV-discordant couples. Only women from Sub-Saharan Africa are included. If studies included sites outside of Sub-Saharan Africa, only women from the Sub-Saharan African sites were included in the meta-analysis.

**3.2. Exposures and outcomes**

**3.2.1. Exposure variable**

The primary exposure of interest is hormonal contraceptive use, specifically the use of combined OCs (including estrogen plus progestin), and the injectable progestins DMPA (150 mg administered IM every 3 months) and Net-En (200 mg administered IM every 2 months). OCs, DMPA and Net-En use will be measured as time-varying exposures during a number of consecutive visit segments (e.g. every 3 months).

Our primary comparison group will be women not using hormonal contraceptives during the time period of interest and will be comprised of women not using any modern contraceptive method, women using condoms (either consistently or inconsistently), sterilized women, and women using intrauterine devices (IUDs) or diaphragms.

**3.2.2. Outcomes**

The primary outcome variable is incident HIV infection. This will be defined as a new HIV infection following a visit where the participant was HIV negative. The criteria for HIV diagnoses are those defined by the investigators of the individual studies and are typically based on a positive ELISA/rapid test confirmed by a positive Western blot or HIV PCR test. The midpoint between the last negative and first positive HIV test will be used as the estimated HIV infection date.

**3.3 Censoring**

Study participants will be censored at the time they report using a non-study method (such as the progestin-only pill or high dose COCs) or at the end of the study or at the last follow-up visit.

**3.4. Assessment of the risk of bias in individual studies**

All studies included in the IPD meta-analysis will be cohort studies or randomized trials.

Knowledge of methodological features that can bias study results is important for interpreting the findings from individual studies and how the differences between studies might contribute to heterogeneity in study results. Unlike meta-analyses based on aggregated data, which generally rely only on information within published papers, an in-depth assessment of the risk of bias in individual studies will be possible. The assessment will make use of the individual participant data, study protocols, clinical study reports, available publications and communications with the study investigators. Additional factors can be assessed, such as whether those lost to follow-up were systematically different to those who remained in the study.

This process has already been completed for the original ten studies included in the VPRP IPD meta-analysis but will be revisited to include issues related to the assessment of exposure variables (hormonal contraception). A full assessment will be conducted for the new studies added to this meta-analysis. Using the study protocol, published articles and data sent by the studies, two reviewers will assess independently the following items (no score will be used) that has been adapted from the Strengthening the Reporting of Observational Studies (STROBE) statement and checklist (20):

1. How much data on main exposures and outcomes are missing?

2. Was the level of retention high in the study (>80% at 12 months)?

3. How frequently was hormonal contraception (main exposure) and HIV infection (outcome) measured?

4. Were exposures categories (e.g. DMPA vs. Net-En injections) clearly defined and measured?

5. Were important covariates measured in the study?

6. Were there large numbers of women (>10%) in the non-hormonal comparison group?

**4. DATA COLLECTION AND MANAGEMENT**

We will follow the data format of the VPRP for all datasets to be included in the meta-analysis. We will assign a unique number to each study and each woman. We will retain a list with our IPD meta-analysis identification numbers and the original study identification numbers and study number in case clarification about specific variables is needed. Three levels of variables will be created; study-level variables, individual-level variables and visit-level variables.

- **Study-level variables include**: country, study design and population group(s); HIV incidence; aims of the study; recruitment period; study duration; frequency of follow up; planned follow-up duration for each woman; definitions of primary and secondary study outcomes; diagnostic procedures, and methodological quality.
- **Individual-level information includes**: unique identification number; date of birth; educational level; employment status; religion; socio-economic indicators; number of births; number of lifetime and recent partners; relationship and cohabitation status with main partner; date of final follow up.
- **Visit-level information includes**: date of study visits; attendance at scheduled follow-up; HC use; pregnancy status, vaginal practices; numbers and type of sexual partners; frequency of coital acts; commercial sex; condom use; information on partner risk, HIV, HSV-2, and STI/RTI infection status.

We will not request any information that could identify an individual woman.

**5. ANALYSIS**

The following sections and Table 1 describe the statistical methods that will be used to investigate the objectives and hypotheses in Section 2. We will use descriptive statistics and statistical tests to examine between-study heterogeneity and univariable and multivariable methods for the analysis of IPD.

**5.1 Descriptive Analysis**

We will conduct descriptive analyses before proceeding to meta-analyses to gain a detailed understanding of the data received within and between studies. Descriptive statistics will be used to summarize study participant characteristics (i.e. socio-demographic characteristics, HIV risk behaviors, STI/HIV status and study duration etc.) by each study and overall.

Categorical variables or continuous variables that have been categorized will be summarized by frequencies and percentages and analyzed using Cochran-Mantel-Haenszel tests across study groups and sites. Data recorded for continuous variables will be summarized by medians and ranges and analyzed by Wilcoxon Mann Whitney tests among the study groups and/or sites.

**5.2 Assessment of confounding**

Assessment of confounding is a critical aspect of this meta-analysis as it clear that women that choose different methods of contraception likely differ in respect to sexual behavior, condom use, etc. For this reason having adequate information on possible key confounding variables as part of study datasets is part of the study inclusion criteria and also is part of our assessment of bias which guides some sensitivity analyses.

In addition to the possible confounding factors that have been pre-specified by researchers (age, marital status/living with partner, condom use, number of sexual partners), the following factors will be examined, if information is available in all/most datasets:

Demographic and sexual behavioral variables related to the individual participant:

- Recent sexual behavior (concurrent sex partners, coital frequency);
- Study site/region

Partner-related variables representing a woman’s risk of sexual exposure to HIV:

- Primary partner’s medical history (e.g. circumcision status);
- Primary partner’s age
- Primary partner’s recent sexual behavior (number of sex partners, partner had other partners, had commercial sex, condom use);

Variables that attempt to measure a woman’s susceptibility to HIV infection:

- Sexual and health behaviors (anal sex, oral sex, vaginal practices);
- Reproductive health factors (parity, pregnancy history and status, lactation status);
- Physical exam variables (cervical ectopy, genital epithelial findings)
- Presence of cervical infections (CT, GC) and vaginal infections (bacterial vaginosis)

We will measure and examine for confounding by factors that either increase a woman’s risk of exposure or susceptibility to HIV infection, and which may also be differentially distributed among contraceptive use exposure groups.

We will identify the set of potential confounding factors found in most/all studies. Each potential confounding factor will be included and excluded in the Cox Proportional Hazards model along with HC exposures and the pre-specified covariates (above) to evaluate the change in the hazard ratios associated with HC exposure. If a greater than 10% change occurs in the hazard ratios for the contraceptive exposure variables results with inclusion of the potential confounder, the variable will be included in the final models of COC, DMPA and NET-EN use and HIV infection.

**5.3. Effect modification**

Young age and HSV-2 infection play an important role in HIV acquisition as illustrated in many studies (3, 9, 12, 18, 19). Because it is plausible that young age and HSV-2 infection status modify potential relationships between hormonal contraception and HIV infection, we have specifically included them in the study objectives and will investigate whether use of various hormonal contraceptives (OCs, DMPA and Net-En) increase the risk of HIV acquisition among different age groups of women and among women infected and not infected with HSV-2 infection. We will evaluate their effect of modification between hormonal contraception and HIV acquisition by using Cox proportional hazard models for each individual study and overall. The modification effect for age and HSV-2 infection status will be evaluated in separate models. If the interaction effect is significant at the p<0.05 level, it indicates that the interactions exist between the contraceptive exposure variable and the effect modifiers.

**5.4. Pregnancy**

The issue of how to analyze women who become pregnant during the follow-up period is complicated since pregnancies that occurred are directly related to HC exposure and clearly have an impact on subsequent HC exposure. In addition, some studies censored women from their studies after a pregnancy occurred. Several analyses will be conducted. As the primary analysis, we will censor study participants after their last non-pregnant visit (i.e., after the last visit before they become pregnant). We note however, that since pregnancy is an outcome directly related to HC exposure and may be related to HIV acquisition, excluding or censoring women with pregnancy might bias the relationship between HC exposure and HIV acquisition. Furthermore, we will conduct two sensitivity analyses for evaluating the impact of the pregnancy on the HC-HIV acquisition relationship. First, we will include pregnancy as a time-varying covariate in a Cox regression analysis model. Second, we will conduct an analysis where we exclude women who ever became pregnant during any of the studies from the analysis population and reanalyze the data. The person-time between these sensitivity analyses and the primary data analysis method will also be evaluated.

**5.5 Meta-analysis**

We will conduct meta-analysis of individual participant data using both one- and two-stage meta-analysis approaches (21). Each method has advantages and disadvantages. When both methods are used, they can provide valuable complementary information.

**5.5.1 Two-stage meta-analysis**

We will use two-stage meta-analysis as our primary approach to examine the overall association between HC exposures and HIV acquisition. Using this method, participants in each study are compared directly only with other participants in the same study. The two-stage method is well suited to assess between study heterogeneity. While the method is less suitable for identifying prognostic factors (due to the different sets of covariates measured across datasets), it is possible to assess effect modification.

In the first stage, IPD in each study are analyzed separately to obtain summary statistics. We will use univariable Cox regression models to estimate the hazard ratio for the association between HC exposures and HIV acquisition. We will then construct multivariable models for each study to control for confounding factors identified in section 5.2.

If appropriate, the second stage is to use standard meta-analysis techniques to combine the summary measures to give an overall estimate of effect across studies. The decision to combine results and conduct further analyses using random and/or fixed effect models depends on the level of between study heterogeneity.

**5.5.1.a Heterogeneity Assessment**

Marked heterogeneity between studies may make it inappropriate to calculate an overall summary measure of effect, but exploring this heterogeneity can provide valuable insights. First, we will examine between-study heterogeneity in associations between exposures and outcomes visually using forest plots of the summary estimates. We will use two statistics to measure the degree of heterogeneity in this meta-analysis: 1) the Q-statistic for which a p-value <0.10 will be interpreted as statistical evidence of heterogeneity (exceeding what would be expected by chance); 2) the I^2^ statistic and its 95% confidence interval, which describes the percentage of total variation across studies due to heterogeneity other than chance (22).

We will use the I^2^ statistic to classify the degree of between-study heterogeneity into low heterogeneity, mild or moderate heterogeneity and high heterogeneity. If we find low between-study heterogeneity, no further investigation of heterogeneity will be done. Otherwise, we will examine potential reasons for between-study heterogeneity using stratification or meta-regression. The outcome of this exploration of heterogeneity will determine decisions about the appropriateness of meta-analysis to pool the effect estimates from the component studies. If the decision is to pool the estimates, random-effect models will be used to incorporate the heterogeneity.

**5.5.2 One-stage meta-analysis**

The one-stage method combines and analyzes data from all studies as if they belong to a single study. Study identity is included in statistical models to take into account the fact that the data are from different studies. This can be seen as a multilevel model, with two levels, which allows the estimation of effects of interest in relation to both study-level and patient-level covariates. The one-stage method might not be a valid option if there is marked heterogeneity between the studies.

We will first use stratified Cox regression to examine the overall unadjusted associations. This allows the survival curve to differ arbitrarily between studies, while assuming that hazard ratios for the exposures of interest are the same across studies. For time-to-event data, the (log)-hazard ratio will be used as the measure of treatment effect. We will then construct multivariable Cox models (23) stratified by study that includes confounding factors identified in section 5.2 and summarize the HC exposure effects on HIV acquisition.

**5.5.3 Study limitations and sensitivity analyses**

Though this meta-analysis provides high quality individual participant data and valuable information about the exposure effect of HC on HIV acquisition, our approach has some limitations. First, the validity of the results of the meta-analysis is dependent on the quality of the individual component studies. While the IPD meta-analysis can help avoid problems associated with the analyses and reporting of the component studies, it cannot eliminate bias due to their study design or conduct. For example, systematic differences between participants’ characteristics in the HC exposure groups may occur in the component studies resulting in imbalances in prognostic factors associated with HIV acquisition. In addition, due to the variety of study designs, not all the subgroups and potential confounding variables are available in all component studies. It is also possible that we may be unable to recode variables appropriately across studies. Together, these factors could potentially limit our ability to control for confounding and residual confounding could occur. In addition there are less Net-En data available in the component studies than there are data for OCs and DMPA.

We will perform the following sensitivity analyses to examine the robustness of the results of our meta-analyses (Table 3):

1. Based on the quality of the HC exposure data, we will stratify and analyze data/studies by the following criteria
   - where data/studies cannot distinguish between progestin-only injectables and implants,
   - or where data/studies cannot distinguish between Net-En and DMPA injectable contraceptives,
   - or where data/studies cannot distinguish between COC and POP oral contraceptives.
2. Based on the assessment of the risk of bias of the component study – stratify studies with and without an identified risk of bias (e.g. studies that have retention rates lower than 80% at one year).
3. Based on HC exposure switching – censoring participants at the visit before the first contraceptive method switch.
4. Based on pregnancy status
   - Include pregnancy as a time-varying covariate (i.e. not censoring women at the time of pregnancy)
   - exclude women who are ever pregnant from the analysis
5. Based on key confounding or risk factor variables – stratify studies that do not measure key confounders in a time-varying manner (e.g. participant risk factors, condom use).

Finally, in consideration of the described potential limitations of our study, we will interpret the results of the meta-analyses cautiously.

**5.5.4 Supplementary analyses**

We will apply the approaches mentioned above to conduct the following supplementary analyses (see Table 3) based on:

1. Condom use: We will conduct an analysis of our primary objectives among the subset of women that report no condom use during the study period.
2. High vs. low incidence study populations: We will conduct a stratified analysis of the HC and HIV association based on stratifying studies with high vs. low HIV incidence in the non-hormonal contraceptive group (using the median as the cutpoint).

**REFERENCES**

1. Low N, Chersich MF, Schmidlin K et al. Intravaginal Practices, Bacterial Vaginosis, and HIV Infection in women: Individual Participant Data Meta-analysis. *PLoS Med* 2011; 8(2): e1000416. Doi:10.1371/journal.pmed.1000416.
2. Ungchusak K, Rehle T, Thammapronpilap P et al. Determinant of HIV infection among female commercial sex workers in northeastern Thailand: results from a longitudinal study. *J Acquir Immune Defic Syndr Hum Retrovirol* 1996; 12: 500-507.
3. Kumwenda JJ, Makanani B, Taulo F et al. Natural history and risk factors associated with early and established HIV type 1 infection among reproductive-age women in Malawi. *CID* 2008; 46: 1913-1920.
4. Feldblum PJ, Lie CC, Weaver MA et al. Baseline factors associated with incident HIV and STI in four microbicide trials. *Sexually Transmitted Diseases* 2010; 37: 594-601.
5. Heffron R, Donnell D, Rees H et al. for the Partners in Prevention HSV/HIV Transmission Study Team. Use of hormonal contraceptives and risk of HIV-1 transmission: a prospective cohort study. *The Lancet Infectious Diseases* 2011; published online Oct 4, 2011. DOI:10:1016/S1473-3099(11)70247-X.
6. Bultreys M, Chao A, Habimana P et al. Incident HIV-1 infection in a cohort of young women in Butare, Rwanda. *AIDS* 1994; 8: 1585-1591.
7. Kleinschmidt I, Rees H, Delany S et al. Injectable progestin contraceptive use and risk of HIV infection in a South African family planning cohort. *Contraception* 2007; 75: 461-467.
8. Baeten JM, Benki S, Chohan V et al. Hormonal contraceptive use, herpes simplex virus infection, and risk of HIV-1 acquisition among Kenyan women. *AIDS* 2007; 21: 1771-1777.
9. Watson-Jones D, Baisley K, Weiss HA et al. Risk factors for HIV incidence in women participating in an HSV suppressive treatment trial in Tanzania. *AIDS* 2009; 23: 415-422.
10. Kilmarx PH, Limpakarnjanarat K, Mastro TD et al. HIV-1 seroconversion in a prospective study of female sex workers in northern Thailand: continued high incidence among brothel-based women. *AIDS* 1998; 12: 1889-1898.
11. Morrison CS, Chen PL, Kwok C, et al. Hormonal contraception and HIV acquisition: reanalysis using marginal structural modeling. *AIDS* 2010; 24: 1778-1781.
12. Morrison CS, Skoler-Karpoff S, Kowk C, et al. Hormonal Contraception and the risk of HIV acquisition among women in South Africa. *AIDS* 2012; 26: 497-504.
13. Wand H and Ramjee G. The effects of injectable hormonal contraceptives on HIV seroconversion and on sexually transmitted infections. *AIDS* 2012; 26: 375-380.
14. Myer L, Denny L, Wright TC et al. Prospective study of hormonal contraception and women’s risk of HIV infection in South Africa. *International Journal of Epidemiology* 2007; 36: 166-174.
15. Reid SE, Dai JY, Wang J et al. Pregnancy, contraceptive use, and HIV acquisition in HPTN 039: relevance for HIV prevention trials among African women. *JAIDS* 2010; 53: 606-613.
16. Kiddugavu M, Makumbi F, Wawer M et al. Hormonal contraceptive use and HIV-1 infection in a population-based cohort in Rakai, Uganda. *AIDS* 2003; 17: 233-240.
17. Kapiga SH, Lyamuya EF, Lwihula GK et al. The incidence of HIV infection among women using family planning methods in Dar es Salaam, Tanzania. *AIDS* 1998; 12: 75-84.
18. Morrsion CS, Richardson BA, Mmiro F, et al. Hormonal contraception and the risk of HIV acquisition. *AIDS* 2007; 21: 85-95.
19. Brown JM, Wald A, Hubbard A, et al. Incident and prevalent herpes simplex virus type 2 infection increases risk of HIV acquisition among young women in Uganda and Zimbabwe. *AIDS* 2007; 21: 1515-1523.
20. von Elm E, Altman DG, Egger M, et al. The strengthening the reporting of observational studies in epidemiology (STROBE) statement: guidelines for reporting observational studies. *PLoS Med* 2007; 4: e296. 10.1371/journal.pmed.0040296.
21. Bowden J, Tierney JF, Simmonds M, et al. Individual patient data meta-analysis of time-to-event outcomes: one-stage versus two-stage approaches for estimating the hazard ratio under a random effects model. *Research Synthesis Methods* 2011; 2: 150–162. doi: 10.1002/jrsm.45.
22. Higgins J, Thompson S. Quantifying heterogeneity in a meta-analysis. *Statistics in Medicine* 2002; 21(11):1539-58.
23. Smith C, Williamson P, Marson A. Investigating heterogeneity in an individual patient data meta-analysis of time to event outcomes. *Statistics in Medicine* 2005; 24:1307-19.

**Table 1. Vaginal Practices Research Partnership Studies (VPRP)**

| Study  # | Country | PI | Study Population | Study Duration | Follow-up | Included  *n* | Incident HIV Infections  *n* | HIV Incidence  (per 100 wy) |
| --- | --- | --- | --- | --- | --- | --- | --- | --- |
| 1 | Kenya (Mombasa) | McClelland | Female sex workers | Not fixed | Monthly | 1,270 | 164 | 11.3 |
| 2 | South Africa | Myer | Women not screened for cervical cancer | 6-36 months | 3 months | 4,160 | 68 | 1.7 |
| 3 | Uganda | Morrison | Women attending RH clinics | 15-24 months | 3 months | 2,201 | 63 | 1.6 |
| 3 | Zimbabwe | Morrison | Women attending RH clinics | 15-24 months | 3 months | 2,248 | 153 | 4.2 |
| 4 | Kenya | Kaul | Female sex workers | 24 months | 6 months | 414 | 30 | 4.9 |
| 5 | Tanzania | Francis | Women working in bars | 12 months | 3 months | 978 | 23 | 2.8 |
| 6 | Tanzania | Watson-Jones | Women working in bars | 30 months | 3 months | 781 | 45 | 3.4 |
| 7 | Zimbabwe  (MIRA) | van der Straten, Padian | Sexually active women | 12-24 months | 3 months | 2,455 | 114 | 2.7 |
| 7 | South Africa  (MIRA) | van der Straten, Padian | Sexually active women | 12-24 months | 3 months | 2,493 | 195 | ~5.1 |
| 9 | South Africa  (RHRU) | Delaney-Moretlwe | Women attending clinics | 12 months | 6 months | 694 | 20 | 3.4 |
| 10 | South Africa | McGrath | Women attending clinics | 12 months | 3 months | 261 | 29 | 15.1 |
| 11 | Malawi | Kumwenda, Brown | Women attending clinics | 9 months | 3 months | 993 | 33 | 4.9 |
| 11 | Zimbabwe | Kumwenda,  Brown | Women attending clinics | 9 months | 3 months | 526 | 19 | 5.2 |

**Table 2. Additional studies to be added to VPRP dataset for HC-HIV IPD Meta-analysis**

| Study  # | Country | PI | Study Population | Study Duration | Follow-up | Included  *n* | Incident HIV Infections  *n* | HIV  Incidence  (per 100 wy) |
| --- | --- | --- | --- | --- | --- | --- | --- | --- |
| 8 | South Africa  (PALESA) | Kleinschmidt, Rees | Women attending RH clinics | 12 months | 3 months | 551 | 23 | 4.7 |
| 12 | South Africa  (Carraguard) | Skoler-Karpoff | Sexually active women | 9-24 months | 3 months | 5,567 | 270 | 3.7 |
| 13 | Uganda | Hayes,  Francis | Sex workers | 12 months | 3 months | 448 | 18 | 4.7 |
| 14 | Tanzania | Hayes, Francis | High risk women | 12 months | 3 months | 876 | 33 | 4.1 |
| 15 | East/Southern Africa  (partners in prev) | Heffron, Baeten | Sexually active women | 24 months | 3 months | 1,314 | 73 | 4.1 |
| 16 | East/Southern  Africa  (MDP301) | McCormack | Sexually active women | 12-24 months | monthly | 8,859 | 419 | 4.3 |
| 17 | South Africa (CAPRISA) | Salim Karim | Sexually active women | Mean 18 months | monthly | 444 | 60 | 9.1 |
| 18 | East/Southern Africa (FEM-PrEP) | Van Damme | Sexually active women | Up to 60 weeks | monthly | 1058 | 35 | 5.0 |

***Total of 38,591 participants and 1,887 incident HIV infections across all included studies**

**Table 3. Planned Analyses and Analysis Populations**

| **Analysis Population** | **PLANNED ANALYSES** | | | | | |
| --- | --- | --- | --- | --- | --- | --- |
|  | **Descriptive** | **Unadjusted** | **Primary** | **Secondary** | **Sensitivity** | **Supplementary** |
| All age groups | √ | √ | √ |  |  |  |
| Young age population |  |  | √ |  |  |  |
| Older age population  (> 24 years) |  |  | √ |  |  |  |
| Women by HSV-2 status |  |  | √ |  |  |  |
| HC groups comparison (DMPA vs. COC, etc.) |  |  |  | √ |  |  |
| Censor studies with high risk of bias |  |  |  |  | √ |  |
| No missing key confounding variables |  |  |  |  | √ |  |
| Censor at first HC switch |  |  |  |  | √ |  |
| Exclude poor HC data |  |  |  |  | √ |  |
| Include pregnancy as time-varying covariate |  |  |  |  | √ |  |
| Exclude pregnant women |  |  |  |  | √ |  |
| Women reporting no condom use |  |  |  |  |  | √ |
| Women in high vs. low HIV incidence populations |  |  |  |  |  | √ |
