# Supplementary material for: Hormonal Contraception and the Risk of HIV Acquisition: An Individual Participant Data Meta-analysis
Source: PLoS Med. 2015 Jan 22;12(1):e1001778. doi: 10.1371/journal.pmed.1001778 (PMC4303292; doi:10.1371/journal.pmed.1001778)
Supplement: S2 Text — (DOCX) [file pmed.1001778.s007.docx]

**FHI 360**

**Hormonal Contraception and the Risk of HIV Acquisition: An Individual Participant Data Meta-Analysis**

**(HC-HIV Meta-analysis)**

**(NICHD award number R21HD069192/ FHI 360 Study # 10263)**

**Detailed Statistical Analysis Plan**

# INTRODUCTION

This study is a meta-analysis of individual participant data (IPD) from 18 prospective longitudinal studies. The main aim of the IPD meta-analysis is to determine whether hormonal contraception, including combined oral contraceptives (COCs) and the progestin-only injectables DMPA and Net-En increase women’s risk of acquiring HIV infection. We will compare the rate of HIV acquisition among women who use COCs, DMPA and Net-En with the rate among women who do not use hormonal contraception. This statistical analysis plan is an extended version of the statistical analysis plan from *HC-HIV IPD Meta-Analysis Protocol - Statistical Analysis Plan 0.95*. This analysis plan provides details on analysis strategy, variable construction and statistical testing procedures.

# STUDY OBJECTIVES

We will investigate the following objectives:

1. To determine whether use of different hormonal contraceptives (COCs, DMPA and Net-En, separately) increases the risk of HIV acquisition compared to women not using hormonal contraception.
   1. To determine whether use of different hormonal contraceptives (COCs, DMPA and Net-En, separately) increases the risk of HIV acquisition among young women (ages 15-24 years).
   2. To determine whether use of different hormonal contraceptives (COCs, DMPA and Net-En, separately) increases the risk of HIV acquisition among older women (ages 25-49 years).
   3. To evaluate whether HSV-2 infection status (across both age groups) modifies the effect of hormonal contraception (DMPA, COCs, and Net-En, separately) on the risk of HIV acquisition and to determine whether different hormonal contraceptives increase the risk of HIV acquisition among HSV-2 negative and HSV-2 positive women.
2. To compare the risks of HIV acquisition among the three hormonal contraceptive groups (DMPA, OC and Net-En users).

# EXPOSURES AND OUTCOMES

## Exposure variable

The primary exposure of interest is hormonal contraceptive use, specifically the use of low-dose COCs (including estrogen plus progestin), and the injectable progestins DMPA (150 mg depot medroxyprogesterone acetate administered IM every 3 months) and Net-En (200 mg norethisterone enanthate administered IM every 2 months). COCs, DMPA and Net-En use will be measured as time-varying exposures during a number of consecutive visit segments (e.g. every 3 months). No washout period will be considered for DMPA and Net-En use because most studies do not collect specific injection dates.

Our primary comparison group, a non-hormonal (NH) group, will be women not using hormonal contraceptives during the interval between study visits and will be comprised of women not using any modern contraceptive method, women using condoms (either consistently or inconsistently), sterilized women, and women using intrauterine devices (IUDs) or diaphragms.

## Outcomes

The primary outcome variable is incident HIV infection. This will be defined as a new HIV infection following a visit where the participant was HIV negative. The criteria for HIV diagnoses are those defined by the investigators of the individual studies and are typically based on a positive ELISA/rapid test confirmed by a positive Western blot or HIV PCR test. The midpoint between the last negative and first positive HIV test will be used as the estimated HIV infection date.

## Censoring

Study participants will be censored at the time they report using a non-study method (such as the progestin-only pill or high dose COCs) or at the end of the study or at the last follow-up visit before 30 months of the study. Though some studies did not distinguish low dose COCs from the progestin-only pill or high dose COCs, we will conduct sensitivity analyses based on those studies that have and have not specified low dose COC use during the study period.

## Data imputation

In principle, missing data won’t be imputed for the data analysis. However, due to the nature of the study design and time-varying contraceptive use, we will impute contraceptive use between visits by carrying forward across visits contraceptive data if the data was collected through questions concerning method use at the current visit. Similarly, we will impute the contraceptive use between visits by carrying backward across visits contraceptive data if the data was collected with questions concerning contraceptive method use in the past. Similarly, this imputation approach will apply to other key confounders such as condom use and high-risk sexual behaviors.

# ANALYSIS METHODS

The following sections describe the statistical methods that will be used to investigate the objectives. We will use descriptive statistics and statistical tests to examine between-study heterogeneity and univariable and multivariable methods for the analysis of IPD.

## Descriptive Analysis

We will conduct descriptive analyses before proceeding to meta-analyses to gain a detailed understanding of the data received within and between studies. Descriptive statistics will be used to summarize study participant baseline characteristics for each study, region and overall. Those baseline characteristics will include

- Socio-demographic characteristics
  - Age
  - Education
  - Marital status/living with partner
  - Employment (Yes/No)
  - Sexual worker (Yes/No) or exchange sex for money/gifts
- Number of live births
- Condom use
  - Any condom use (any condom use, no condom use, no sex)
  - Condom use for primary partner
  - Condom use for other partner(s)
  - Condom use for unspecified partner(s)
  - Condom use with last sex
- Contraceptive use (COC, OC unspecified, DMPA, Net-en, injectable unspecified, implant, non-hormonal)
- Sexual behavior
  - Age at first sex
  - Coital frequency in past month
  - Anal sex (Yes/No)
  - Number of lifetime partners
  - Number of partners in past 3 months
  - New partner
  - Concurrent partner
- Primary partner information
  - Partner had HIV
  - Partner had another partner
  - Partner had nights
- STI/RTIs
  - Chlamydia infection
  - Gonococcal infection
  - Trichomonas infection
  - BV
  - Vaginal discharge/cervicitis
  - Candida vaginitis
  - HSV-2
  - Genital ulcer (clinician diagnosed)
- Vaginal practice
  - no practice/water (fingers only)
  - soap
  - cloth/tissue/cotton wool
  - other practices besides soap or cloth/tissue/cotton wool
- Self-reported symptoms
  - Itching
  - Abnormal discharge
  - ulcer

Categorical variables or continuous variables that have been categorized will be summarized by frequencies and percentages and analyzed using Cochran-Mantel-Haenszel tests across study groups and sites. Data recorded for continuous variables will be summarized by medians and ranges and analyzed by Wilcoxon Mann Whitney tests among the study groups and/or sites.

## Assessment of confounding

Assessment of confounding is a critical aspect of this meta-analysis as it clear that women that choose different methods of contraception likely differ in respect to sexual behavior, condom use, etc. For this reason having adequate information on possible key confounding variables as part of study datasets is part of the study inclusion criteria and also is part of our assessment of bias, which guides some sensitivity analyses.

In addition to the possible confounding factors that have been pre-specified by researchers (age, marital status/living with partner, condom use, number of sexual partners), the following factors will be examined, if information is available in all/most datasets:

Demographic and sexual behavioral variables related to the individual participant:

- Recent sexual behavior (concurrent sex partners, coital frequency);
- Study site/region

Partner-related variables representing a woman’s risk of sexual exposure to HIV:

- Primary partner’s recent sexual behavior (number of sex partners, partner had other partners, had commercial sex, condom use);

Variables that attempt to measure a woman’s susceptibility to HIV infection:

- Sexual and health behaviors (anal sex, oral sex, vaginal practices);
- Reproductive health factors (parity, pregnancy history and status, lactation status);
- Physical exam variables (cervical ectopy, genital epithelial findings)
- Presence of cervical infections (CT, GC) and vaginal infections (bacterial vaginosis, trichomoniasis, vulvovaginal candidiasis)

We will measure and examine for confounding by factors that either increase a woman’s risk of exposure or susceptibility to HIV infection, and which may also be differentially distributed among contraceptive use exposure groups. Each potential confounding factor will be included and excluded in the Cox Proportional Hazards model for each individual study along with HC exposures and the pre-specified covariates (above) to evaluate the change in the hazard ratios associated with HC exposure. If a greater than 10% change occurs in the hazard ratios for the contraceptive exposure variables results with inclusion of the potential confounder, the variable will be considered for inclusion in the final models of COC, DMPA and NET-EN use and HIV infection in the meta-analysis.

## Effect modification

Though a priori hypotheses about age (younger vs. older) and HSV-2 infection (not infected vs. infected) status will be addressed individually in the meta-analysis, the question of whether age and HSV-2 infection status modify potential relationships between hormonal contraception and HIV infection is still of interest. We will evaluate their modification effect on the hormonal contraception and HIV acquisition relationship by adding an interaction term between age and HC, and between HSV-2 and HC using Cox proportional hazard models for each individual study and overall. The modification effect for age and HSV-2 infection status will be evaluated in separate models. If p<0.05 for the likelihood ratio test comparing models with and without the interaction term, statistical evidence of interaction (i.e. effect modification) between the contraceptive exposure variable and HIV will be concluded.

## Pregnancy

The issue of how to analyze women who become pregnant during the follow-up period is complicated since pregnancies that occurred are directly related to HC exposure and clearly have an impact on subsequent HC exposure. In addition, some studies censored women from their studies after a pregnancy occurred. Several analyses will be conducted. As the primary analysis, we will censor study participants after their last non-pregnant visit (i.e., after the last visit before they become pregnant). We note however, that since pregnancy is an outcome directly related to HC exposure and may be related to HIV acquisition, excluding or censoring women with pregnancy might bias the relationship between HC exposure and HIV acquisition. In addition, we will conduct two sensitivity analyses for evaluating the impact of the pregnancy on the HC-HIV acquisition relationship. First, we will include pregnancy as a time-varying covariate in a Cox regression analysis model. Second, we will conduct an analysis where we exclude women who ever became pregnant during any of the studies from the analysis population and reanalyze the data. The person-time between these sensitivity analyses and the primary data analysis method will also be evaluated.

## Meta-analysis

We will conduct meta-analysis of individual participant data using both one- and two-stage meta-analysis approaches in each age group and combined, and for studies that provided HSV-2 information (1). Each method has advantages and disadvantages. When both methods are used, they can provide valuable complementary information.

We will use two-stage meta-analysis as our primary approach to examine the overall association between HC exposures and HIV acquisition. Using this method, participants in each study are compared directly only with other participants in the same study. The two-stage method is well suited to assess between study heterogeneity. While the method is less suitable for identifying prognostic factors (due to the different sets of covariates measured across datasets), it is possible to assess effect modification.

The one-stage method combines and analyzes data from all studies as if they belong to a single study. Study identity is included in statistical models to take into account the fact that the data are from different studies. The one-stage method might not be a valid option if there is marked heterogeneity between the studies. We will explore the effects of confounding and effect modification using the one-stage method. If there is statistical evidence of confounding, we will include the confounding variables in the two-stage multivariable analysis.

### Two-stage Meta-analysis

**Univariable Analysis**: We will examine the associations between HC exposures and HIV in each study by using Cox proportional hazard regression. The hazard ratio (and 95% CI) will be used as the measure of the HC exposure effect. We will use the two-stage method to examine the association in each study. In the first stage, IPD in each study are analyzed separately to obtain the hazard ratio for each HC exposure vs. non-hormonal group. We then will use the random effects model as the primary method to obtain the weighted combined HC exposure effect on HIV acquisition from all studies. This method assumes that there is a distribution of true hazard ratios. The combined effect therefore represents the mean of the population of true hazard ratios. In addition, we will conduct a sensitivity analysis with the assumption that the true hazard ratio is the same in all studies (using a fixed effect model).

**Multivariable Analysis**: We will conduct two separate multivariable Cox proportional hazard regression analyses for each study controlling for confounding factors. The first multivariable analysis - our primary analysis - will include a common set of confounders that are pre-specified in the study protocol (age, marital status/living with partner, condom use, and number of sexual partners) and/or identified using the one-stage meta-analysis approach for all studies. The second multivariable analysis will include the confounders identified for each study individually, i.e., the best multivariable model based on available data in each individual study. The same random effects model and fixed effect approaches as stated in the univariate analysis will be applied to combine the adjusted hazard ratios to give a weighted overall estimate of the effect of HC exposures on HIV acquisition.

### Heterogeneity Assessment: We will then use those associations to investigate the heterogeneity between studies. First, we will examine between-study heterogeneity in associations between exposures and outcomes visually using forest plots of the summary estimates. We will use two statistics to measure the degree of heterogeneity in this meta-analysis: 1) the Q-statistic for which a p-value <0.10 will be interpreted as statistical evidence of heterogeneity (exceeding what would be expected by chance); 2) the I^2^ statistic and its 95% confidence interval, which describes the percentage of total variation across studies due to heterogeneity other than chance (2).

We will use the I^2^ statistic to classify the degree of between-study heterogeneity into low heterogeneity (I^2^ <50%), mild or moderate heterogeneity (I^2^ between 50-75%), and high heterogeneity (I^2^ > 75%). If we find low between-study heterogeneity, no further investigation of heterogeneity will be done. Otherwise, we will examine potential reasons for between-study heterogeneity using stratification or meta-regression. For example, we will stratify the analysis by study region, participant population (sex worker vs. general population) and HIV incidence in the non-hormonal group. The outcome of this exploration of heterogeneity will determine decisions about the appropriateness of meta-analysis to pool the effect estimates from the component studies.

### One-stage Meta-analysis

We will first use the stratified Cox proportional hazard regression to examine the overall unadjusted associations. This allows the survival curve to differ arbitrarily between studies, while assuming that hazard ratios for the exposures of interest are the same across studies. This result will be compared to the overall effect estimated by the univariable analysis from the two-stage meta-analysis. We will then construct multivariable Cox models (3) stratified by study that include identified confounding factors and/or have been pre-specified in the study protocol (age, marital status/living with partner, condom use, and number of sexual partners), and summarize the HC exposure effects on HIV acquisition. Random effects Cox proportional hazard regressions with studies as clusters will also be conducted for univariate and multivariable analyses. This can be seen as a multilevel model with two levels, which allows the estimation of effects of interest in relation to both study-level and participant-level covariates. The effect of modification between HC and age and HSV-2 infection, respectively, will be examined using the approaches proposed above.

### Sensitivity Analyses

Though this meta-analysis provides high quality individual participant data and valuable information about the exposure effect of HC on HIV acquisition, our approach has some limitations. First, the validity of the results of the meta-analysis is dependent on the risk of bias in the component studies. While the IPD meta-analysis can help avoid problems associated with the analyses and reporting of the component studies, it cannot eliminate bias due to their study design or conduct. For example, systematic differences between participants’ characteristics in the HC exposure groups may occur in the component studies resulting in imbalances in prognostic factors associated with HIV acquisition. In addition, due to the variety of study designs, not all the subgroups and potential confounding variables are available in all component studies. It is also possible that we may be unable to recode variables appropriately across studies. Together, these factors could potentially limit our ability to control for confounding and residual confounding could occur. In addition there are fewer Net-En data available in the component studies than there are data for COCs and DMPA.

We will perform the following sensitivity analyses to examine the robustness of the results of our meta-analyses (Table 1):

1. Based on the risk of bias in HC exposure data, we will stratify and analyze data/studies separately by the following criteria
   1. where data/studies cannot distinguish between progestin-only injectables and implants,
   2. Where data/studies cannot distinguish between Net-En and DMPA injectable contraceptives,
   3. Where data/studies cannot distinguish between COC and POP oral contraceptives.
2. Based on the assessment of the risk of bias of the component study – stratify studies with and without an identified risk of bias (e.g. studies that have retention rates lower than 80% at one year).
3. Based on HC exposure switching – censoring participants at the visit before the first contraceptive method switch.
4. Based on pregnancy status
   1. Include pregnancy as a time-varying covariate (ie. not censoring women at the time of pregnancy)
   2. exclude women who are ever pregnant from the analysis
5. Based on key confounding or risk factor variables – stratify studies that do not measure key confounders in a time-varying manner (e.g. participant risk factors, condom use).

Finally, in consideration of the described potential limitations of our study, we will interpret the results of the meta-analyses cautiously, based on a priori hypotheses.

### Supplementary Analyses

We will apply the approaches mentioned above to conduct the following supplementary analyses (see Table 1) based on:

1. Condom use: We will conduct an analysis of our primary objectives among the subset of women that report no condom use during the study period.
2. High vs. low incidence study populations: We will conduct a stratified analysis of the HC and HIV association based on stratifying studies with high vs. low HIV incidence in the non-hormonal contraceptive group (using the median as the cutpoint).
3. Women by region (Eastern Africa, Southern Africa and South Africa)
4. Age groups among young women: If there are sufficient numbers of HIV cases among 15-24 year old age group, we will conduct an analysis for the primary objectives based on finer age groups: 15-20 years and 21-24 years (versus women > 25 years).

**REFERENCES**

1. Bowden J, Tierney JF, Simmonds M, et al. Individual patient data meta-analysis of time-to-event outcomes: one-stage versus two-stage approaches for estimating the hazard ratio under a random effects model. *Research Synthesis Methods* 2011; 2: 150–162. doi: 10.1002/jrsm.45.
2. Higgins J, Thompson S. Quantifying heterogeneity in a meta-analysis. *Statistics in Medicine* 2002; 21(11):1539-58.
3. Smith C, Williamson P, Marson A. Investigating heterogeneity in an individual patient data meta-analysis of time to event outcomes. *Statistics in Medicine* 2005; 24:1307-19.

**Table 1. Planned Analyses and Analysis Populations**

| **Analysis Population** | **PLANNED ANALYSES** | | | | | |
| --- | --- | --- | --- | --- | --- | --- |
|  | **Descriptive** | **Unadjusted** | **Primary** | **Secondary** | **Sensitivity** | **Supplementary** |
| All age groups | √ | √ | √ |  |  |  |
| Young age population |  |  | √ |  |  |  |
| Older age population  (> 24 years) |  |  | √ |  |  |  |
| Women by HSV-2 status |  |  | √ |  |  |  |
| HC groups comparison (DMPA vs. COC, etc.) |  |  |  | √ |  |  |
| Stratify studies by risk of bias |  |  |  |  | √ |  |
| No missing key confounding variables |  |  |  |  | √ |  |
| Censor at first HC switch |  |  |  |  | √ |  |
| Stratify based on quality of HC data |  |  |  |  | √ |  |
| Include pregnancy as time-varying covariate |  |  |  |  | √ |  |
| Exclude pregnant women |  |  |  |  | √ |  |
| Women reporting no condom use |  |  |  |  |  | √ |
| Women in high vs. low HIV incidence populations |  |  |  |  |  | √ |
| Women among the three regions |  |  |  |  |  | √ |
| Women ages 15-20, 21-24 vs. > 25 years |  |  |  |  |  | √ |
